# Comparative effectiveness and safety of four traditional Chinese medicine injections with invigorating blood circulation, equivalent effect of anticoagulation or antiplatelet in acute myocardial infarction: a Bayesian network meta-analysis

**DOI:** 10.3389/fphar.2024.1400990

**Published:** 2024-08-14

**Authors:** Jiaping Chen, Juju Shang, Hongxu Liu, Xiang Li, Xiaolei Lai, Yan Lou, Huiwen Zhou

**Affiliations:** Department of Cardiology, Beijing Hospital of Traditional Chinese Medicine Affiliated to Capital Medical University, Beijing, China

**Keywords:** Chinese medicine injections, acute myocardial infarction, network meta-analysis, traditional Chinese medicine, invigorate blood circulation

## Abstract

**Background:** Traditional Chinese medicine injections with invigorating blood circulation (TCMI-IBCs), which have been used as antithrombosis therapies, are widely employed by Chinese clinicians as adjuvant therapy for acute myocardial infarction (AMI).

**Objective:** A Bayesian network meta-analysis was conducted to contrast the effectiveness and safety of four TCMI-IBCs in AMI.

**Methods:** Eight Databases were thoroughly searched before 31 December 2023, for randomized controlled trials (RCTs) focusing on the application of TCMI-IBCs combined with conventional treatments (CT) to treat AMI. All-cause mortality (ACM) was the major endpoint. Secondary outcomes included bleeding events, malignant arrhythmia (MA), recurrent myocardial infarction (RMI), left ventricular ejection fraction (LVEF), and adverse events. Stata17.0 and GeMTC software were employed for Bayesian network meta-analysis.

**Results:** A total of 73 eligible RCTs involving 7,504 patients were enrolled. Puerarin injection (PI), Danhong injection (DI), sodium Tanshinone IIA Sulfonate injection (STSI), and Danshen Chuanxiongqin injection (DCI) combined with CT can significantly reduce the occurrence of ACM and improve LVEF in AMI (*P* < 0.05), while without significant impact on bleeding events or MA (*P >* 0.05). STSI + CT would be the optimal treatment strategy in lowering RMI and ACM. DI + CT was the most likely to be the optimal strategy in reducing MA occurrence and improving LVEF. CT was likely the most effective strategy in reducing bleeding events. However, DI + CT exhibited the least favorable safety.

**Conclusion:** TCMI-IBCs + CT had potential benefits in the treatment of AMI. STSI + CT showed the most favorable performance in treating AMI, followed by DI combined with CT.

**Systematic Review Registration:** https://www.crd.york.ac.uk/PROSPERO/display_record.php?RecordID=384067, identifier CRD42022384067.

## Graphical Abstract

**Figure:**
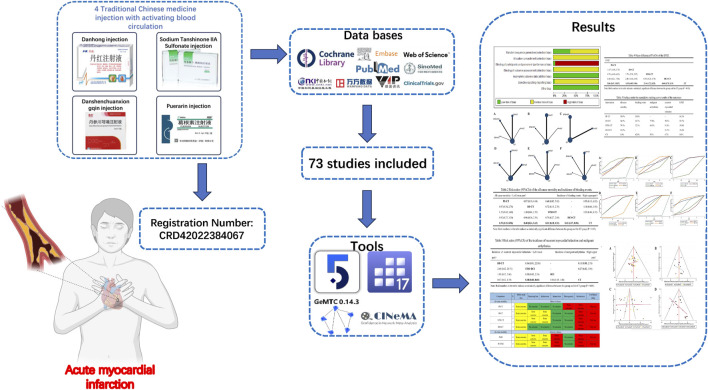

## 1 Introduction

According to the *Global Burden of Disease Report 2020*, ischemic heart disease claimed the lives of 8.95 million people globally (Tsao et al., 2022). Among them, acute myocardial infarction (AMI) is the ischemic heart disease with the highest mortality and disability rate worldwide (Roth et al., 2020), which also occurs in China. Over the past decade, the management of AMI in China has made some progress. However, the *China Cardiovascular Disease and Health Report 2021 Summary* indicates that the overall mortality of AMI patients in China is still increasing, imposing a significant burden on both the economy and healthcare system (The Writing Committee of the Report on Cardiovascular Health and Diseases in China, 2022). The treatment of AMI involves general treatment, reperfusion therapy, as well as pharmacotherapy (anti-myocardial ischemia, antiplatelet, anticoagulation, lipid-lowering, etc.) (Levine et al., 2016; Ibanez et al., 2018; Collet et al., 2021). Early reperfusion therapy can restore blood flow in the occluded vessel, reducing the mortality among AMI patients (Roger et al., 2010; Hall et al., 2016). Nonetheless, there are still several unresolved issues following reperfusion, including decreased myocardial contractility, ventricular arrhythmia, no-reflow phenomenon, and in-stent restenosis (Eeckhout and Kern, 2001; Thiele et al., 2017; de Waha et al., 2018; Pantea-Rosan et al., 2020). These complications have a significant impact on patients’ prognosis (Heusch and Gersh, 2017). Exploring alternative, effective treatment approaches is still necessary.

Classified as “True heartache,” AMI is thought to be a consequence of the obstruction of cardiac blood vessels, according to traditional Chinese medicine theory. Promoting blood circulation and removing blood stasis is the focus of the treatment principle (Doctor Society of Integrative Medicine, 2018). In China, traditional Chinese Medicine injections (TCMIs) have gradually emerged as an adjunctive therapy for AMI (Spatz et al., 2018). TCMIs with invigorating blood circulation (TCMI-IBCs), which have equivalent effects of anticoagulant and antiplatelet, have been demonstrated in several trials to reduce the mortality and the risk of in-stent restenosis in AMI patients (Liu et al., 2012; Liu et al., 2014). However, the evidence regarding the effectiveness and safety of these TCMI-IBCs in terms of methodology and evidence-based evaluation is limited (Liu et al., 2011; Shang et al., 2012).

Previous systematic reviews (Liao et al., 2015; Hua et al., 2021; Wang and Xie, 2021) have assessed the effectiveness of TCMI-IBCs in the management of AMI, but the comparative effectiveness and safety among different TCMI-IBCs remain uncertain. Network meta-analysis is an analytical approach derived from routine meta-analysis, enabling the comparison and ranking of the efficacy of various interventions for one disease (Wu et al., 2017). This study utilized the data platform of the “National Drug Standards of China State Food and Drug Administration” (https://www.nmpa.gov.cn/) to identify TCMI-IBCs indicated for AMI treatment. Four TCMI-IBCs were discovered through the search: Puerarin Injection (PI), Danhong Injection (DI), Sodium Tanshinone IIA Sulfonate Injection (STSI), and Danshen Chuanxiongqin Injection (DCI). Networks meta-analysis was employed to contrast the clinical effectiveness and safety of four TCMI-IBCs combined with conventional treatment (CT) for AMI, thus to provide more reference for clinical treatment.

## 2 Methods

This study was conducted following the protocol registered with PROSPERO (Protocol number: CRD42022384067). The Preferred Reporting Items for Systematic Reviews and Meta-analyses (PRISMA) guidelines (Hutton et al., 2015) were employed to conduct our network meta-analysis, as seen in Supplementary Figure. To ensure accurate reporting of four TCMIs in this analysis, we adhered to the guidelines established in the consensus statement on the Phytochemical Characterization of Medicinal Plant extract (ConPhyMP) (Supplementary Tables 1–4).

### 2.1 Search strategy

We searched PubMed, Cochrane Library, Embase, the Web of Science, Chinese Biological Medicine Database (CBM), Wanfang Database, China National Knowledge Infrastructure (CNKI), and Chinese Scientific Journal Database (VIP) without language limitation published before 31 December 2023. Other online search resources included *ClinicalTrials.gov* and proceedings of major cardiovascular conferences. A subset of Chinese and English journals related to AMI was also manually searched. Search terms include acute myocardial infarction, puerarin injection, Danhong injection, Sodium Tanshinone IIA Sulfonate injection, Danshen Chuanxiongqin injection, and randomized controlled trial. The search strategy is shown in Supplementary Tables 5.

### 2.2 Study selection

Study selection was independently performed by two researchers (JPC and HWZ) and disagreements were resolved by discussion with the third researcher (HXL).

The inclusion criteria are as follows: 1. Participants: All study participants need to meet the diagnostic criteria of AMI (Gao, 2001; Thygesen et al., 2018); 2. Interventions: The control group received CT or CT combined with a placebo. CT included general treatment (monitoring of vital signs, alleviating symptoms, etc.), medication (antiplatelet, lipid-lowering, anticoagulant, etc.), and reperfusion therapy (percutaneous coronary intervention (PCI), thrombolysis, and coronary artery bypass surgery). Based on the control group, the intervention group was combined with one of these four TCMI-IBCs (including PI, DI, STSI, and PI), and the treatment dose and duration of the above drugs need to conform to the drug instructions; 3. Outcome measures: At least one outcome was reported. 1) Primary outcome: all-cause mortality (ACM) (all-cause deaths during hospitalization); 2) Secondary outcomes: Any bleeding events, malignant arrhythmias (MA), recurrent myocardial infarction (RMI), left ventricular ejection fraction (LVEF), and adverse events; 4. Study design: randomized controlled trials (RCTs), blinded or not, were included.

Studies that meet the following criteria will be excluded: 1. Full text is unavailable, data cannot be extracted or is incomplete, and there are obvious problems with the data; 2. Case report, review, conference literature, theoretical discussion, and experience summary; 3. Patients’ baseline information was inconsistent; 4. Repeated publications (retain the most comprehensive one).

### 2.3 Data extraction

Data extraction was completed by two researchers (XL and XLL) independently and cross-checked. When necessary data were missing in the literature, we attempted to contact the authors to complete the data. The extracted data include the first author, publication year, sample size, gender composition, age, treatment course, intervention measures, outcomes, and the information of quality assessment of RCTs. If the result index was measured at various times, the data measured at the last moment were included.

### 2.4 Risk of bias and quality of evidence assessment

Two investigators (YL and JJS) independently evaluated the risk of bias and the quality of evidence. Through discourse with the third researcher (HXL), discrepancies were resolved. The Cochrane Risk of Bias Tool was employed to evaluate the risk of bias of included RCTs, based on the following items: random sequence generation, allocation concealment, blinding of participants and personnel, blinding of outcome assessment, incomplete outcome data, selective reporting, and other bias. The CINeMA online application (https://cinema.ispm.unibe.ch/#) was employed to evaluate the quality of the evidence. Different from the GRADE method, CINeMA comprehensively evaluates the results of network meta-analysis in six areas: intra-study bias, interstudy bias, indirectness, inaccuracy, heterogeneity, and inconsistency (Nikolakopoulou et al., 2020).

### 2.5 Data synthesis and statistical analysis

In this study, the analysis results for LVEF are reported as mean differences (MD), while odds ratios (OR) are used to represent the analysis results for binary variables such as ACM, bleeding events, MA, RMI, and adverse events. Each effect size is accompanied by a 95% confidence interval (*CI*). A network meta-analysis was performed using Stata17.0 to visualize the network relationships among different intervention measures. If a closed loop appears in the evidence network plot, the consistency between indirect and direct comparisons is assessed using inconsistency testing. In this study, no closed loops were observed in any of the outcomes, indicating that a consistency model was selected for the network meta-analysis. A “calibration-comparison” funnel plot was created to determine the presence or absence of small sample effects and evaluate publication bias. GeMTC software was employed to conduct a Bayesian model network meta-analysis, beginning with four Markov chains. The initial value was set to 2.5, and a pre-iteration of 20,000 times was conducted for annealing. Iteration was then continued for 50,000 times to achieve model convergence. Model convergence was deemed satisfactory when the potential scale reduction factor (PSRF) approached 1. Otherwise, additional iterations were performed. Using the surface under the cumulative ranking curve (SUCRA) ranked the efficacy of different TCMI-IBCs on each outcome (Yi et al., 2015). Sensitivity analysis was conducted by setting the criterion of ≥80 participants to assess the robustness of the study results. Additionally, we performed conventional meta-analyses using the DerSimonian-Laird random-effects model and the Mantel-Haenszel fixed-effects model to provide direct estimates.

## 3 Results

### 3.1 Study selection

**FIGURE 1:**
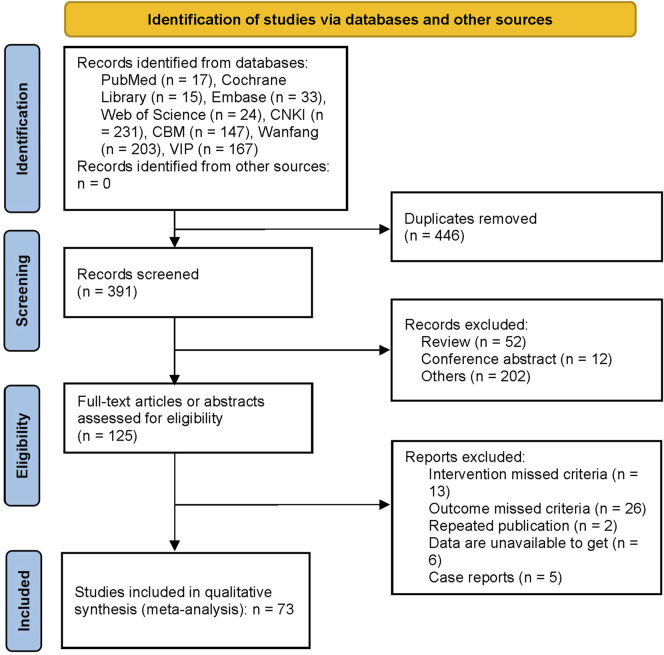
Flow chart of the study selection. The initial review yielded 837 articles, leaving 391 articles after removing duplicates. After selection based on title or abstract, 125 articles were screened for full text review. Of these, 13 articles were combined with oral commercial Chinese polyherbal preparation, 26 articles’ outcomes missed criteria, two articles had duplicate publications, six articles had unextractable data, and five articles were case reports.

The initial review yielded 837 articles, leaving 391 articles after removing duplicates. After selection based on title or abstract, 125 articles were subjected to full text review. Of these, 13 articles were combined with oral commercial Chinese polyherbal preparation (CCPP), 26 articles’ outcomes failed to meet criteria, two articles had duplicate publications, six articles had unquantifiable data, and five articles were case reports. At last, 73 articles were included, 70 of which were in Chinese and three in English, all of which had been conducted in mainland China. The literature screening process is shown in Figure 1.

### 3.2 Study characteristics

**FIGURE 2:**
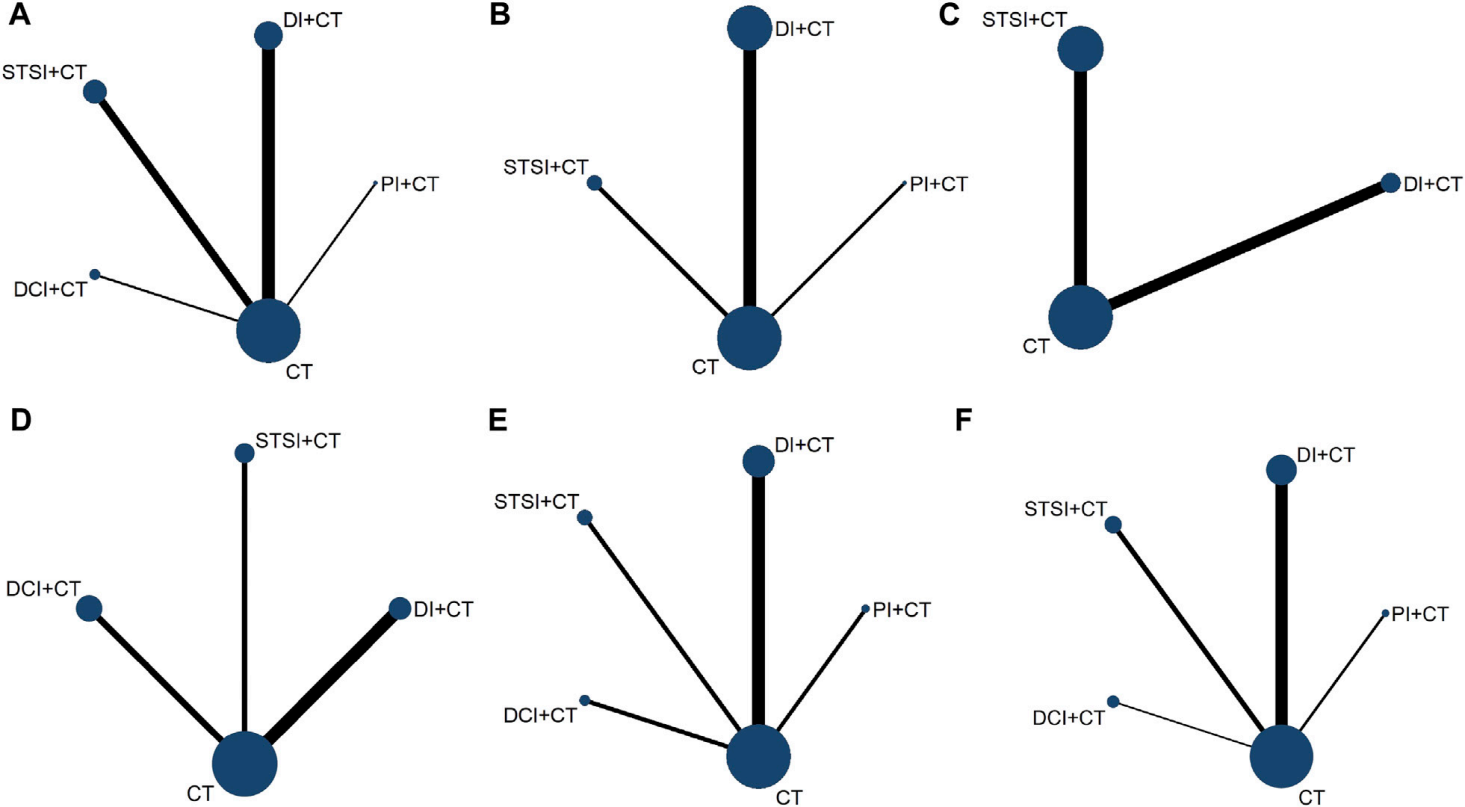
Network graph of the outcomes. This figure presents the evidence network diagram for each outcome measure, and none of them formed a closed loop. **(A)**, 38 RCTs involving four TCMI-IBCs (PI, DI, STSI, and DCI) reported the ACM; **(B)**, 17 RCTs involving three TCMI-IBCs (PI, DI, and STSI) reported the incidence of bleeding events; **(C)**, MA was reported in 10 RCTs involving two TCMI-IBCs (DI and STSI); **(D)**, RMI was reported in 13 RCTs involving three TCMI-IBCs (DI, STSI, and DCI); **(E)**, 27 RCTs involving four TCMI-IBCs (PI, DI, STSI, and DCI) reported LVEF; **(F)**, 23 RCTs involving four TCMI-IBCs (PI, DI, STSI, and DCI) reported adverse events. Each node represents an intervention, and the line thickness between nodes represents the number of studies included between the two interventions. The Stata17.0 was used to visualize the network relationships among different intervention measures.

The 73 RCTs (Lu et al., 2001; Li et al., 2002; Gu and Wang, 2004; Xiao et al., 2004; Qi et al., 2006; Chen, 2007; Liu and Zhong, 2008; Qu, 2008; Yu et al., 2008; Gao, 2009; Gui et al., 2009; Chen et al., 2010; Han, 2010; Zhou et al., 2010; Du and Wang, 2011; Hao and Ren, 2011; Sun, 2011; Feng and He, 2012; Han et al., 2012; Huang, 2012; Zhang, 2012; Zhao, 2012; Chen and Liu, 2013; Kong et al., 2013; Ren and Jia, 2013; Liu and Mao, 2014; Qin et al., 2014; Zhou, 2014; Bin and Shaopeng, 2015; Fan and Yang, 2015; Liu, 2015; Wang and Jie, 2015; Xu et al., 2015; Yang et al., 2015; Huang, 2016; Ji et al., 2016; Liu et al., 2016; Sun et al., 2016; Ceng et al., 2017; Fan and Chen, 2017; Xu and Liu, 2017; Zhang, 2017; Zhou, 2017; Fu Z. G. et al., 2018; Luo R. X., 2018; Luo X. Y., 2018; Hu, 2018; Lin et al., 2018; Miao et al., 2018; Zhang et al., 2018; Cao and Di, 2019; Huo et al., 2019; Jin et al., 2019; Mao et al., 2019; Qi et al., 2019; Shen et al., 2019; You, 2019; Zhang et al., 2019; Lan et al., 2020; Li and Xu, 2020; Liu et al., 2020; Qin et al., 2020; Zhiyong et al., 2020; Chen L. F. et al., 2021; Chen Q. et al., 2021; Cui et al., 2021; Feng, 2021; Lu et al., 2021; Zhang, 2021; Dong and Fang, 2022; Song, 2022) included were two-arm trials with a total of 7,504 patients. All the included RCTs described the baseline information, ensuring comparability among the groups. In all studies, the follow-up duration spanned from 2 weeks to 12 months, respectively. The details of the study characteristics are depicted in Table 1; Supplementary Tables 6. Figure 2 presents the evidence network diagram for each outcome, and none of them formed a closed loop, therefore, the assumption of consistency was not tested.

**TABLE 1:** Characteristics of the included studies.

Study ID	N (E/C)	Sex (M/F)	Age(Y) (E/C)	STEMI, Non-STEMI (E/C)	I(E)	I (C)	Course	Follow-up	Outcomes
Cao and Di (2019)	63/63	72/54	55.7 ± 5.2/58.5 ± 5.8	NR	STSI 40 mg Qd + Am	Am	3M	NR	I
Chen et al. (2021a)	40/40	45/35	71.98 ± 2.86/72.31 ± 2.89	NR	DI 30 mL Qd + TT	TT	14d	NR	V
Chen, (2007)	33/31	49/15	54 ± 4/52 ± 6	NR	STSI 40 mg Qd + P + Ac + B + N	P + Ac + B + N	14d	NR	I
Chen et al. (2010)	29/30	43/16	62 ± 5/65 ± 4	All STEMI	DI 20 mL Qd + P + Ac + PCI	P + Ac + PCI	14d	NR	V
Chen and Liu (2013)	39/36	40/35	52.3 ± 3.6/53.1 ± 2.9	All Non-STEMI	DI 30 mL Qd + P + Ac + S + B + N	P + Ac + S + B + N	14d	NR	VI
Chen et al. (2021b)	46/47	56/37	62.9 ± 9.5/63.2 ± 8.5	All STEMI	DI 40 mL Qd + P + Ac + S + B + N + PCI	P + Ac + S + B + N + PCI	2 h	1M	I; III; IV
Cui et al. (2021)	45/45	46/44	57.53 ± 3.35/56.35 ± 3.23	NR	DI 30 mL Qd + A + S + TT	A + S + TT	60d	6M	IV
Dong and Fang (2022)	49/49	55/39	47.61 ± 1.94/47.82 ± 1.72	NR	PI 200 mg Qd + P + N + Am + TT	P + N + Am + TT	24d	NR	V; VI
Du and Wang (2011)	26/22	NR	59 ± 12	NR	STSI 80 mg Qd + P + PCI	P + PCI	14d	NR	I; III
Fan and Yang (2015)	47/42	49/38	59.7 ± 5.6/58.9 ± 4.4	NR	STSI 60 mg Qd + P + Ac + TT	P + Ac + TT	7d	NR	V
Fan and Chen (2017)	49/49	53/45	53.6 ± 3.9/57.6 ± 3.9	NR	PI 300 mg Qd + P + Ac + TT	P + Ac + TT+	14d	NR	V; VI
Feng and He (2012)	50/50	56/44	61.5 ± 5.2/61.1 ± 5.3	NR	STSI 60 mg Qd + P + Ac + N + TT	P + Ac + N + TT	7d	NR	III
Feng and He (2012)	79/78	82/95	60.19 ± 1.38/60.25 ± 1.21	All STEMI	DI 40 mL Qd + P + Ac + PCI	P + Ac + PCI	7d	3M	V; VI
Fu et al. (2018a)	48/48	60/36	58.9 ± 4.9/58.3 ± 5.2	All STEMI	DI 30 mL Qd + P + Ac + TT	P + Ac + TT	7d	12M	I; II
Gao (2009)	60/60	82/44	57.9/56.6	NR	DI 30 mL Qd + P + N + TT	P + N + TT	14d	NR	I
Gao (2015)	40/40	62/18	59.1 ± 2.86/59.6 ± 3.2	NR	STSI 80 mg Qd + P + Ac + S + B + A + TT + PCI	P + Ac + S + B + A + TT + PCI	14d	NR	III; V;VI
Gu and Wang (2004)	34/31	49/16	61.5	NR	PI 500 mg Qd + P + Ac + A + N + TT	P + Ac + A + N + TT	21d	NR	V
Gui et al. (2009)	26/22	Sep-39	56.38 ± 8.86/55.86 ± 10.94	NR	DI 20 mL Qd + P + Ac + S + N + TT	P + Ac + S + N + TT	14d	NR	I; II; IV
Han (2010)	50/50	69/31	53.0 ± 16.2/52.5 ± 16.5	NR	DI 20 mL Qd + P + Ac + TT	P + Ac + TT	7d	NR	I; II
Han et al. (2012)	76/58	74/60	55.6 ± 12.5/51.8 ± 13.6	NR	DI 20 mL Qd + P + Ac + TT	P + Ac + TT	14d	NR	I; II
Hao and Ren (2011)	60/60	79/41	59.2 ± 11.2	NR	DI 30 mL Qd + PAc + B + A + TT	P + Ac + B + A + TT	7d	NR	I; II
Hu (2018)	44/43	48/39	60.71 ± 5.51/59.60 ± 5.62	NR	DI 30 mL Qd + P + Ac + S + B + N + TT	P + Ac + S + B + N + TT	15d	NR	II; VI
Huang (2012)	42/43	50/35	60.2 ± 6.7/61.5 ± 6.4	NR	STSI 400 mg Qd + P + Ac + B + N	P + Ac + B + N	7d	NR	I
Huang (2016)	50/50	63/37	52.13 ± 8.59/53.61 ± 9.47	NR	DCI 10 mL Qd + P + Ac + S + N	P + Ac + S + N	28d	NR	V
Huo et al. (2019)	40/40	54/26	62 ± 4/61 ± 6	NR	DI Qd + P + Ac + S + B + A + N	P + Ac + S + B + A + N	14d	NR	V
Ji et al. (2016)	54/54	74/34	62.3 ± 6.5/61.8 ± 6.7	All Non-STEMI	DI 40 mL Qd + P + Ac + S + N	P + Ac + S + N	28d	NR	II
Jia (2010)	48/50	56/42	66 ± 9/67 ± 8	NR	STSI 60 mL Qd + P + Ac + B + A + N	P + Ac + B + A + N	28d	NR	II
Jin et al. (2019)	57/60	77/40	57.7 ± 5.49/57.4 ± 6.37	NR	DI 40 mL Qd + P + Ac + B + A + N	P + Ac + B + A + N	14d	NR	V
Kong et al. (2013)	48/42	48/42	66.2 ± 12.0/65.8 ± 13.1	NR	STSI 40 mg Qd + Am	Am	14d	NR	I
Li et al. (2002)	30/30	41/19	52.8 ± 8.5/53.6 ± 7.8	NR	PI 500 mg Qd + P + Ac + TT	P + Ac + TT	15d	NR	I; II
Li et al. (2019)	45/45	43/47	60.14 ± 4.81/60.17 ± 2.85	All STEMI	DI 30 mL Qd + P + PCI	P + PCI	7d	NR	III; VI
Li and Xu (2020)	86/80	85/81	59.25 ± 6.69/58.75 ± 5.68	All STEMI	DI 4 mL Qd + P + Ac + S + PCI	P + Ac + S + PCI	14d	NR	V; VI
Bin and Shaopeng (2015)	30/30	44/16	55.6 ± 2.3/56.7 ± 3.4	NR	DCI 10 mL Qd + P + S + B + A + N	P + S + B + A + N	14d	NR	V
Liu and Zhong (2008)	23/23	28/18	53.11 ± 7.31/51.86 ± 7.38	NR	PI 0.4 g Qd + P + Ac + N + TT	P + Ac + N + TT	14d	NR	I; II
Liu et al. (2014)	80/80	112/48	62.8 ± 8.2/63.1 ± 8.6	NR	STSI 60 mg Bid + P + Ac + TT	P + Ac + TT	1d	NR	III
Liu (2015)	40/40	49/31	60 ± 5.5/61 ± 6.5	NR	DI 20 mL Qd + Ac + TT	Ac + TT	3d	NR	I
Liu et al. (2016)	49/49	56/42	56.9 ± 4.7/57.1 ± 4.8	NR	STSI 40 mg Qd + Am	Am	10d	NR	I
Liu et al. (2020)	61/61	70/52	58.97 ± 6.52/59.32 ± 5.87	All STEMI	DCI 10 mL Qd + P + S + PCI	P + S + PCI	14d	14d	V; VI
Lu et al. (2021)	100/100	115/85	58.06 ± 4.84/58.13 ± 5.16	NR	STSI 80 mg Qd + P + S + PCI	P + S + PCI	7d	12M	I; III; IV
Luo (2018a)	43/43	47/39	57.85 ± 4.61/58.01 ± 5.02	NR	STSI 80 mg Qd + P + PCI	P + PCI	7d	NR	IV; V
Luo (2018b)	54/54	55/53	57.12 ± 5.33/56.23 ± 5.48	NR	STSI 40mgQd + Am	Am	24d	NR	I
Lu et al. (2001)	32/28	42/18	61.6 ± 9.6/60.8 ± 8.8)	NR	PI 400 mg Qd + P + Ac + PCI	P + Ac + PCI	21d	NR	V
Mao et al. (2019)	50/51	51/50	68.19 ± 9.67/68.63 ± 10.23	NR	STSI 80 mg Qd + P + S + B + A	P + S + B + A	7d	6M	I; IV
Miao et al. (2018)	44/44	47/41	59.67 ± 8.14/60.01 ± 8.27	NR	DCI 10 mL Qd + P + B + A + Am	P + B + A + Am	14d	1M	V
Qi et al. (2006)	48/46	65/29	65.22 ± 6.98/62.11 ± 10.25	NR	STSI 80 mg Qd + P + Ac + N	P + Ac + N	14d	NR	I
Qin et al. (2014)	56/56	61/51	52.31 ± 11.24/55.12 ± 10.52	All STEMI	DI 40 mL Qd + P + Ac + S + B + A + PCI	P + Ac + S + B + A + PCI	7d	NR	II; V
Qin et al. (2020)	53/53	63/43	71.43 ± 6.12/70.51 ± 6.09	NR	STSI 40 mg Qd + P + S + TT	P + S + TT	14d	NR	II; V;VI
Qu (2008)	26/26	37/15	60.2 ± 5.3/60.7 ± 5.2	NR	PI 500 mg Qd + B + A + N + TT	B + A + N + TT	28d	NR	I
Ren and Jia (2013)	20/20	27/13	63.1 ± 8.3	NR	DI 40 mL Qd + P + Ac + S + B + A + N	P + Ac + S + B + A + N	14d	14d	I; IV
Shen et al. (2019)	100/100	117/83	59.65 ± 3.79/59.82 ± 3.70	All STEMI	DI 40 mL Qd + P + PCI	P + PCI	14d	NR	II; V
Song (2022)	99/99	108/90	62.19 ± 5.02/62.52 ± 4.78	NR	STSI 80 mg Qd + P + T	P + T	14d	NR	II; V;VI
Sun (2011)	72/64	76/60	56.34/57.82	All Non-STEMI	STSI 80 mg + P + N	P + N	14d	NR	III
Sun et al. (2016)	60/60	64/56	62.7 ± 11.9/62.7 ± 4.2	All STEMI	DCI 15 mL Tid + P + PCI	P + PCI	6d	NR	I; IV
Wang and Jie (2015)	30/30	36/25	64.7 ± 5.6/64.2 ± 5.1	NR	DI 20 mL Qd + P + Ac + S + B + A + N + TT	P + Ac + S + B + A + N + TT	14d	NR	I; III
Xiao et al. (2004)	31/30	43/18	61.3/60.2	NR	PI 500 mg Qd + B + A + N + TT	B + A + N + TT	14d	NR	I
Xu et al. (2015)	36/35	49/22	64 ± 12	NR	DI 40 mL Qd + P + S + B + A + N	P + S + B + A + N	14d	NR	I; VI
Xu and Liu (2017)	42/42	55/49	71.4 ± 5.8/72.3 ± 6.2	NR	DCI 10 mL Qd + P + S + B + A + PCI	P + S + B + A + PCI	7d	NR	V
Yang et al. (2015)	28/29	40/17	64 ± 12.3/65 ± 11.7	All STEMI	DI 40 mL Qd + P + S + A + N	P + S + A + N	10d	NR	I; VI
You (2019)	57/53	95/15	56.8 ± 8.9/55.4 ± 9.5	All STEMI	DI 40 mL Qd + PCI	PCI	4-6d	6M	I
You (2019)	62/57	103/16	58.1 ± 9.9/58.0 ± 9.7	All STEMI	DI 40mlQd + P + Ac + S + PCI	P + Ac + S + PCI	7d	6M	I; V;VI
Yu et al. (2008)	46/50	66/30	63 ± 6.96/62.11 ± 10.26	NR	STSI 80 mg Qd + P + Ac + S + N + TT	P + Ac + S + N + TT	14d	NR	I
Ceng et al. (2017)	60/60	74/46	65.13 ± 2.38/64.38 ± 2.12	All STEMI	DI 40 mL Qd + P + Ac + B + N	P + Ac + B + N	14d	6M	I; IV; V
Zhang (2012)	30/30	39/21	56.9 ± 5.62/56.67 ± 6.41	NR	DI 30 mL Qd + P + Ac + S + B + A + TT	P + Ac + S + B + A + TT	7d	NR	II; III
Zhang (2017)	50/50	48/52	65.23 ± 2.14	NR	STSI 40 mg Qd + Am	Am	14d	NR	I
Zhang et al. (2018)	55/55	68/42	61.9 ± 4.9/62.5 ± 4.2	All Non-STEMI	DI 20 mL Qd + P + Ac + S + N	P + Ac + S + N	14d	NR	II
Zhang et al. (2019)	40/40	46/34	69.88 ± 3.16/68.92 ± 3.08	All STEMI	DI 30 mL Qd + P + Ac + S	P + Ac + S	7d	NR	I
Zhang (2021)	50/50	55/45	60.23 ± 6.54/60.45 ± 6.56	NR	DI 30 mL Qd + P + Ac + S + N + TT	P + Ac + S + N + TT	14d	NR	V
Zhao (2012)	30/26	32/24	60.0 ± 9.0/58.0 ± 9.0	NR	DI 30 mL Qd + TT	TT	14d	NR	I; IV
Zhou et al. (2010)	33/32	35/30	64.5 ± 6.3/62.5 ± 8.4	NR	DI 30 mL Qd + P + Ac + S + B + A + N + PCI	P + Ac + S + B + A + N + PCI	14d	NR	V
Zhou (2014)	100/100	146/54	55.9 ± 1.48/56.0 ± 1.51	All STEMI	DCI 10 mL Qd + P + Ac + B + N	P + Ac + B + N	NR	NR	I; IV; VI
Zhou (2017)	42/42	54/30	67.3 ± 5.8/68.1 ± 5.9	All STEMI	DCI 10 mL Tid + P + PCI	P + PCI	6d	NR	I; IV
Lin et al. (2018)	70/60	NR	75 ± 9/74 ± 8	All STEMI	DCI 10 mL Qd + P + PCI	P + PCI	7d	6M	II; IV
Zhiyong et al. (2020)	74/74	82/66	56.28 ± 7.43/56.73 ± 7.85	NR	DCI 10 mg Qd + P + S + B + A + N + Am	P + S + B + A + N + Am	14d	1M	I; VI

Note: N, number; E, experimental group; C, control group; M, male; F, female; Y, years old; STEMI, ST segment elevation myocardial infarction; Non-STEMI, non-ST segment elevation myocardial infarction; I, intervention measures; d, day; M, month; NR, not report; PI, puerarin injection; DI, danhong injection; STSI, Sodium Tanshinone IIA sulfonate injection; DCI, danshen chuanxiongqin injection; Tid, 3 times a day; Bid, twice a day; Qd, once a day; A, angiotensin converting enzyme inhibitors or angiotensin receptor antagonists; B, beta-blockers; N, nitrate esters; P, antiplatelet aggregation drugs; S, statins; T, trimetazidine; TT, thrombolytic therapy; Am, amiodarone; PCI, percutaneous coronary intervention; Ac, anticoagulation; I, all-cause mortality. II, bleeding events; III, malignant arrhythmia; IV, recurrent myocardial infarction; V, LVEF; VI, adverse reactions.

### 3.3 Quality evaluation

**FIGURE 3:**
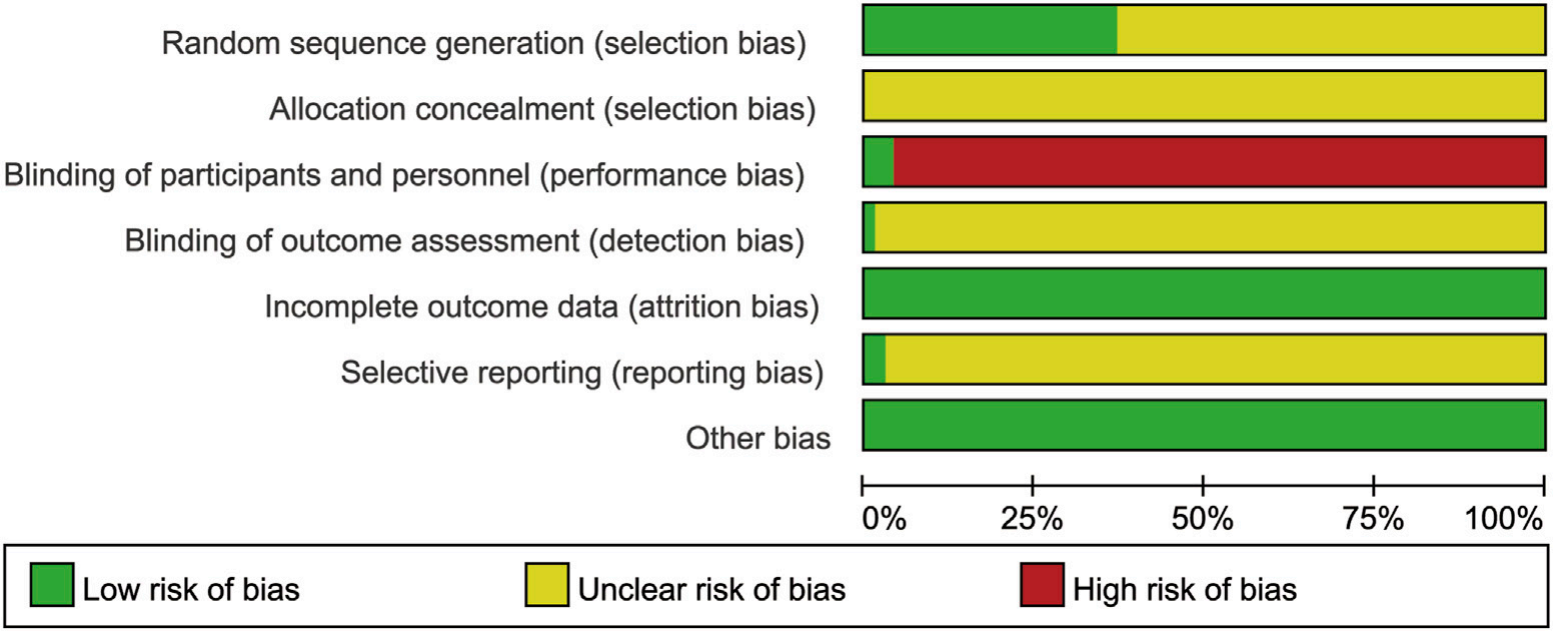
Risk-of-bias graph. For randomization, three studies used stratified randomization, two studies used computer randomization, one study used a coin toss, and 21 studies used a random number table. The risk of the above studies was considered low, while the remaining studies did not describe specific random methods and were rated as unclear. None of the included studies reported allocation concealment, so selection bias was rated as unclear. Three studies were double-blinded, the remaining studies did not mention the blinding method and were judged to be high-risk as it was difficult to apply blinding according to specific treatment measures. One study blinded the outcome evaluators and was judged to be low-risk. The remaining studies did not mention the blinded outcome evaluation and were judged to be unclear. All included RCTs had complete data, so they were considered to be low-risk. Taking into account the inability to acquire a complete implementation scheme, the risk of reporting bias was considered unclear, except two RCTs. For other bias, no significant bias was observed in all studies, so it was considered to be low risk.

For randomization, three studies (Lin et al., 2018; Qi et al., 2019; You, 2019) used stratified randomization, two studies (Zhou, 2017; Mao et al., 2019) used computer randomization, one study (Jin et al., 2019) used a coin toss, and 21 studies (Chen et al., 2010; Jia, 2010; Bin and Shaopeng, 2015; Fan and Yang, 2015; Yang et al., 2015; Huang, 2016; Liu et al., 2016; Ceng et al., 2017; Xu and Liu, 2017; Luo R. X., 2018; Hu, 2018; Zhang et al., 2018; Li and Xu, 2020; Liu et al., 2020; Zhiyong et al., 2020; Chen L. F. et al., 2021; Feng, 2021; Lu et al., 2021; Zhang, 2021; Dong and Fang, 2022; Song, 2022) used a random number table. The risk of the above studies was considered low, while the remaining studies did not describe specific random methods and were rated as unclear. None of the included studies reported allocation concealment, so selection bias was rated as unclear. Three studies (Gui et al., 2009; Liu et al., 2016; Qi et al., 2019) were double-blinded, the remaining studies did not mention the blinding method and were judged to be high-risk as it was difficult to apply blinding according to specific treatment measures. One study (Mao et al., 2019) blinded the outcome evaluators and was judged to be low-risk. The remaining studies did not mention the blinded outcome evaluation and were judged to be unclear. All included RCTs had complete data, so they were considered to be low-risk. Taking into account the inability to acquire a complete implementation scheme, the risk of reporting bias was considered unclear, except two RCTs (Mao et al., 2019; You, 2019). For other bias, no significant bias was observed in all studies, so it was considered to be low risk. The Cochrane bias risk results are shown in Figure 3; Supplementary Figure F1 *.*

### 3.4 Results of the network meta-analysis

#### 3.4.1 ACM

**FIGURE 4:**
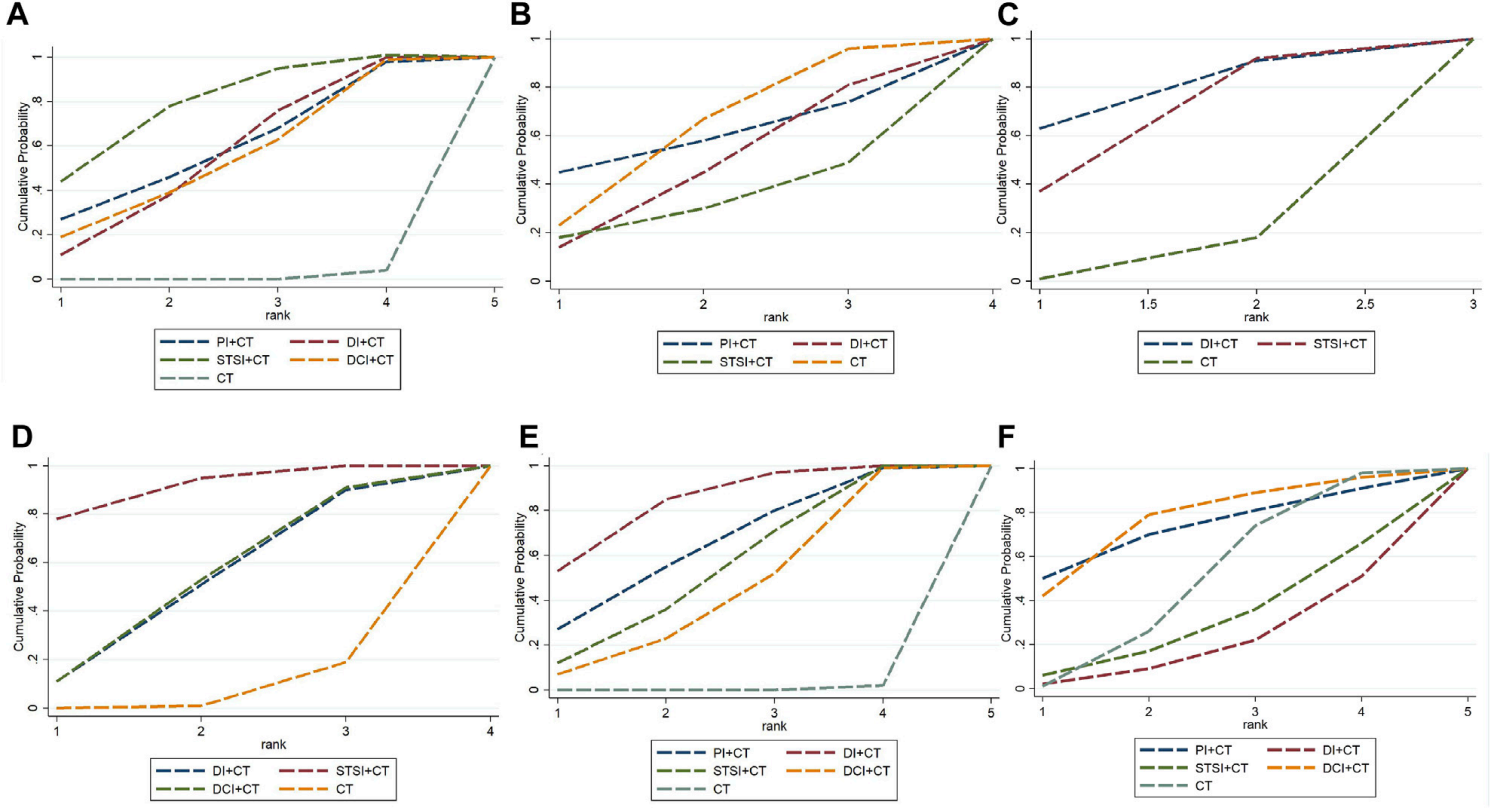
Plot of the surface under the cumulative ranking curves for outcomes. **(A)** In terms of rankings for reducing the incidence of all-cause mortality from the SUCRA analysis, STSI + CT plus ranked the first, followed by PI + CT, DI + CT, DCI + CT, and CT; **(B)** In terms of rankings for reducing the incidence of bleeding events from the SUCRA analysis, CT plus ranked the first, followed by PI + CT, DI + CT, and STSI + CT; **(C)** In terms of rankings for reducing the incidence of malignant arrhythmias from the SUCRA analysis, DI + CT plus ranked the first, followed by STSI + CT and CT; **(D)** In terms of rankings for reducing the incidence of recurrent myocardial infarction from the SUCRA analysis, STSI + CT plus ranked the first, followed by DI + CT, DCI + CT, and CT; **(E)** In terms of rankings for improving LVFE from the SUCRA analysis, DI + CT plus ranked the first, followed by PI + CT, STSI + CT, DCI + CT, and CT; **(F)** In terms of rankings for ruducing the occurrence of adverse reactions from the SUCRA analysis, DCI + CT plus ranked the first, followed by PI + CT, CT, STSI + CT, and DI + CT. Bayesian model network meta-analysis was performed using GeMTC software with four Markov chains as initial values. The initial value was set to 2.5, and a pre-iteration of 20,000 times was conducted for annealing. Iteration was then continued for 50,000 times to achieve model convergence. Model convergence was deemed satisfactory when the potential scale reduction factor (PSRF) approached 1. Otherwise, additional iterations were performed. The intervention effects of different TCMIs on each outcome indicator were ranked using the surface under the cumulative ranking curve (SUCRA).

Thirty-eight RCTs (Li et al., 2002; Xiao et al., 2004; Qi et al., 2006; Chen, 2007; Liu and Zhong, 2008; Qu, 2008; Yu et al., 2008; Gao, 2009; Gui et al., 2009; Han, 2010; Du and Wang, 2011; Hao and Ren, 2011; Han et al., 2012; Huang, 2012; Zhao, 2012; Kong et al., 2013; Ren and Jia, 2013; Zhou, 2014; Liu, 2015; Wang and Jie, 2015; Xu et al., 2015; Yang et al., 2015; Liu et al., 2016; Sun et al., 2016; Ceng et al., 2017; Zhang, 2017; Zhou, 2017; Fu Z. X. et al., 2018; Luo X. Y., 2018; Cao and Di, 2019; Mao et al., 2019; Qi et al., 2019; You, 2019; Zhang et al., 2019; Lan et al., 2020; Zhiyong et al., 2020; Chen L. F. et al., 2021; Lu et al., 2021) involving four TCMI-IBCs (PI, DI, STSI, and DCI) reported the ACM. Compared with CT alone, PI + CT [OR = 0.39, 95%*CI* (0.15, 0.96)], DI + CT [OR = 0.40, 95%*CI* (0.24, 0.63)], STSI + CT [OR = 0.31, 95%*CI* (0.18, 0.51)], and DCI + CT [OR = 0.42, 95%*CI* (0.17, 0.96)] could significantly reduce ACM in AMI patients (Table 2). According to the SUCRA results (Figure 4A; Table 5; Supplementary Tables 7), STSI + CT may be the optimal combination to reduce the occurrence of ACM among the four combinations.

**TABLE 2:** Risk ratios (95%*CI*s) of the all-cause mortality and incidence of bleeding events.

ACM (Left lower part)	Incidence of bleeding events (Right upper part)			
**PI + CT**	0.87 (0.10, 6.04)	0.60 (0.05, 5.82)	-	0.95 (0.13, 6.12)
0.97 (0.34, 2.78)	**DI + CT**	0.72 (0.15, 2.78)	-	1.10 (0.64, 1.88)
1.25 (0.43, 3.69)	1.30 (0.64, 2.55)	**STSI + CT**	-	1.55 (0.44, 6.32)
0.92 (0.27, 3.30)	0.96 (0.36, 2.56)	0.74 (0.27, 2.04)	**DCI + CT**	-
**0.39 (0.15, 0.96)**	**0.40 (0.24, 0.63)**	**0.31 (0.18, 0.51)**	**0.42 (0.17, 0.96)**	**CT**

Note: Bold numbers in the table indicate a statistically significant difference between this group and the CT group (*p* < 0.05).

#### 3.4.2 Incidence of bleeding events

Seventeen RCTs (Li et al., 2002; Liu and Zhong, 2008; Gui et al., 2009; Han, 2010; Jia, 2010; Hao and Ren, 2011; Han et al., 2012; Zhang, 2012; Qin et al., 2014; Ji et al., 2016; Fu Z. G. et al., 2018; Hu, 2018; Lin et al., 2018; Zhang et al., 2018; Shen et al., 2019; Qin et al., 2020; Song, 2022) involving three TCMI-IBCs (PI, DI, and STSI) reported the incidence of bleeding events. In comparison to CT alone, the combination of TCMI-IBCs had no significant effect on reducing the occurrence of bleeding events in AMI (Table 2). The results of SUCRA suggested that CT may be the best strategy to reduce the occurrence of bleeding events (Figure 4B; Table 5; Supplementary Tables 8).

#### 3.4.3 MA

MA was reported in 10 RCTs (Du and Wang, 2011; Sun, 2011; Feng and He, 2012; Zhang, 2012; Liu and Mao, 2014; Wang and Jie, 2015; Li et al., 2019; Lan et al., 2020; Chen L. F. et al., 2021; Lu et al., 2021) involving two TCMI-IBCs (DI and STSI). Compared with CT alone, the combination of TCMI-IBCs had no significant effect on MA in AMI patients (Table 3). According to SUCRA results (Figure 4C; Table 5; Supplementary Tables 9), DI + CT may be the optimal combination in reducing the incidence of MA.

**TABLE 3:** Risk ratios (95%*CI*s) of the incidence of recurrent myocardial infarction and malignant arrhythmias.

RMI (Left lower part)	MA (Right upper part)		
**DI + CT**	0.56 (0.01, 22.01)	-	0.15 (0.00, 2.71)
2.69 (0.42, 20.71)	**STSI + DCI**	-	0.27 (0.02, 1.98)
1.03 (0.17, 5.86)	0.38 (0.05, 2.33)	**DCI**	-
0.47 (0.12, 1.53)	**0.18 (0.03, 0.65)**	0.46 (0.13, 1.48)	**CT**

Note: Bold numbers in the table indicate a statistically significant difference between this group and the CT group (*p* < 0.05).

#### 3.4.4 RMI

RMI was reported in 13 RCTs (Gui et al., 2009; Zhao, 2012; Ren and Jia, 2013; Zhou, 2014; Sun et al., 2016; Ceng et al., 2017; Zhou, 2017; Luo R. X., 2018; Lin et al., 2018; Mao et al., 2019; Chen Q. et al., 2021; Cui et al., 2021; Lu et al., 2021) involving three TCMI-IBCs (DI, STSI, and DCI). Compared with CT alone, STSI combined with CT significantly reduced RMI in AMI patients [OR = 0.18, 95%*CI* (0.03, 0.65)] (Table 3). According to the results of SUCRA (Figure 4D; Table 5; Supplementary Tables 10), STSI + CT may be the best combination in reducing the incidence of RMI.

#### 3.4.5 LVEF

Twenty-seven RCTs (Lu et al., 2001; Gu and Wang, 2004; Chen et al., 2010; Zhou et al., 2010; Qin et al., 2014; Bin and Shaopeng, 2015; Fan and Yang, 2015; Huang, 2016; Ceng et al., 2017; Fan and Chen, 2017; Xu and Liu, 2017; Luo R. X., 2018; Miao et al., 2018; Huo et al., 2019; Jin et al., 2019; Mao et al., 2019; Shen et al., 2019; You, 2019; Lan et al., 2020; Li and Xu, 2020; Liu et al., 2020; Qin et al., 2020; Chen L. F. et al., 2021; Feng, 2021; Zhang, 2021; Dong and Fang, 2022; Song, 2022) involving four TCMI-IBCs (PI, DI, STSI, and DCI) reported LVEF. Compared to CT alone, PI + CT [MD = 5.86, 95%*CI* (1.67, 10.07)], DI + CT [MD = 6.93, 95%*CI* (4.49, 10.07)], STSI + CT [MD = 5.14, 95%*CI* (1.72, 8.55)], and DCI + CT [MD = 4.44, 95%*CI* (0.70, 8.19)] could significantly enhance LVEF in AMI patients (Table 4). According to SUCRA results (Figure 4E; Table 5; Supplementary Tables 11), DI + CT may be the most effective combination in improving LVEF.

**TABLE 4:** Mean difference (95%*CI*s) of the LVEF and adverse events.

RMI (Left lower part)	MA (Right upper part)			
**PI + CT**	0.40 (0.05, 2.69)	2.18 (0.28, 16.64)	1.09 (0.15, 13.20)	0.61 (0.12, 3.65)
−1.07 (−5.91, 3.79)	**DI + CT**	0.85 (0.19, 3.82)	2.93 (0.57, 22.19)	1.57 (0.64, 4.71)
0.71 (−4.69, 6.12)	1.79 (−2.39, 5.97)	**STSI + CT**	2.45 (0.45, 21.26)	1.32 (0.46, 5.20)
1.40 (−4.21, 7.04)	2.48 (−1.96, 6.93)	0.70 (−4.36, 5.78)	**DCI + CT**	0.54 (0.11, 2.21)
**5.86 (1.67, 10.07)**	**6.93 (4.49, 9.36)**	**5.14 (1.72, 8.55)**	**4.44 (0.70, 8.19)**	**CT**

Note: Bold numbers in the table indicate a statistically significant difference between this group and the CT group (*p* < 0.05).

**TABLE 5:** Surface under the cumulative ranking curve results of the outcomes.

Intervention	All-cause mortality(%)	Bleeding events(%)	Malignant arrhythmias(%)	Recurrent myocardial infarction(%)	LVEF(%)	Adverse events(%)
PI + CT	59.8	59.0	-	-	65.3	75.8
DI + CT	56.3	46.7	77.0	50.7	83.7	22.2
STSI + CT	79.3	32.3	64.5	91.0	54.8	35.7
DCI + CT	55.0	-	-	51.7	45.2	77.1
CT	1.0	62.0	9.5	6.7	0.5	39.2

#### 3.4.6 Adverse events

34 RCTs reported adverse events, of which 11 trials (Qu, 2008; Han et al., 2012; Zhang, 2012; Liu, 2015; Wang and Jie, 2015; Huang, 2016; Sun et al., 2016; Luo X. Y., 2018; Miao et al., 2018; Huo et al., 2019; Mao et al., 2019) indicated no adverse events. 23 studies (Gu and Wang, 2004; Qi et al., 2006; Yu et al., 2008; Jia, 2010; Hao and Ren, 2011; Huang, 2012; Zhang, 2012; Chen and Liu, 2013; Zhou, 2014; Xu et al., 2015; Yang et al., 2015; Fan and Chen, 2017; Hu, 2018; Li et al., 2019; Shen et al., 2019; You, 2019; Li and Xu, 2020; Liu et al., 2020; Qin et al., 2020; Feng, 2021; Dong and Fang, 2022; Song, 2022) involving four TCMI-IBCs (PI, DI, STSI, and DCI) described specific adverse events, such as gastrointestinal symptoms, ecchymosis, dizziness, headache, and so on (Table 6). Compared to CT alone, PI + CT [OR = 0.61, 95%*CI* (0.12, 3.65)], DI + CT [OR = 1.57, 95%*CI* (0.64, 4.71)], STSI + CT [OR = 1.32, 95%*CI* (0.46, 5.20)], and DCI + CT [OR = 0.54, 95%*CI* (0.11, 2.21)] could not increase the occurrence of adverse reactions in AMI patients (Table 4). According to SUCRA results (Figure 4F; Table 5; Supplementary Tables 12), DCI + CT exhibited the best favorable safety.

**TABLE 6:** Occurrence of adverse events of TCMI-IBCs.

Intervention measures	PI + CT	DI + CT	STSI + CT	DCI + CT	CT
Number of studies	3	11	6	3	23
Sample size	10	26	35	20	104
Digestive tract symptom	4	7	12	2	27
Ecchymosis	0	3	0	15	17
Dizziness	2	3	0	1	7
Headache	0	2	11	0	8
Hypotension	2	0	3	1	9
Bradycardia	2	2	1	1	19
Palpitation	0	0	2	0	2
Flush face	0	3	4	0	3
Elevated serum creatinine	0	4	0	0	3
Elevated blood uric acid	0	2	0	0	2
Other adverse reactions	0	0	2	0	7

### 3.5 Sensitivity analysis

Fifty-four RCTs (n = 6,092) with ≥80 cases were included in the sensitivity analysis, which revealed that the result of DCI + CT vs*.* CT was different from the original result of ACM, and the result of PI + CT vs*.* CT was different from the original result of LVEF, and no significant deviations were observed for the rest outcomes (Supplementary Tables 16–18). Given that the number of participants may affect the study’s outcomes, the number of patients participating in the trial should be adequately calculated to obtain more reliable results.

### 3.6 Publication bias

**FIGURE 5:**
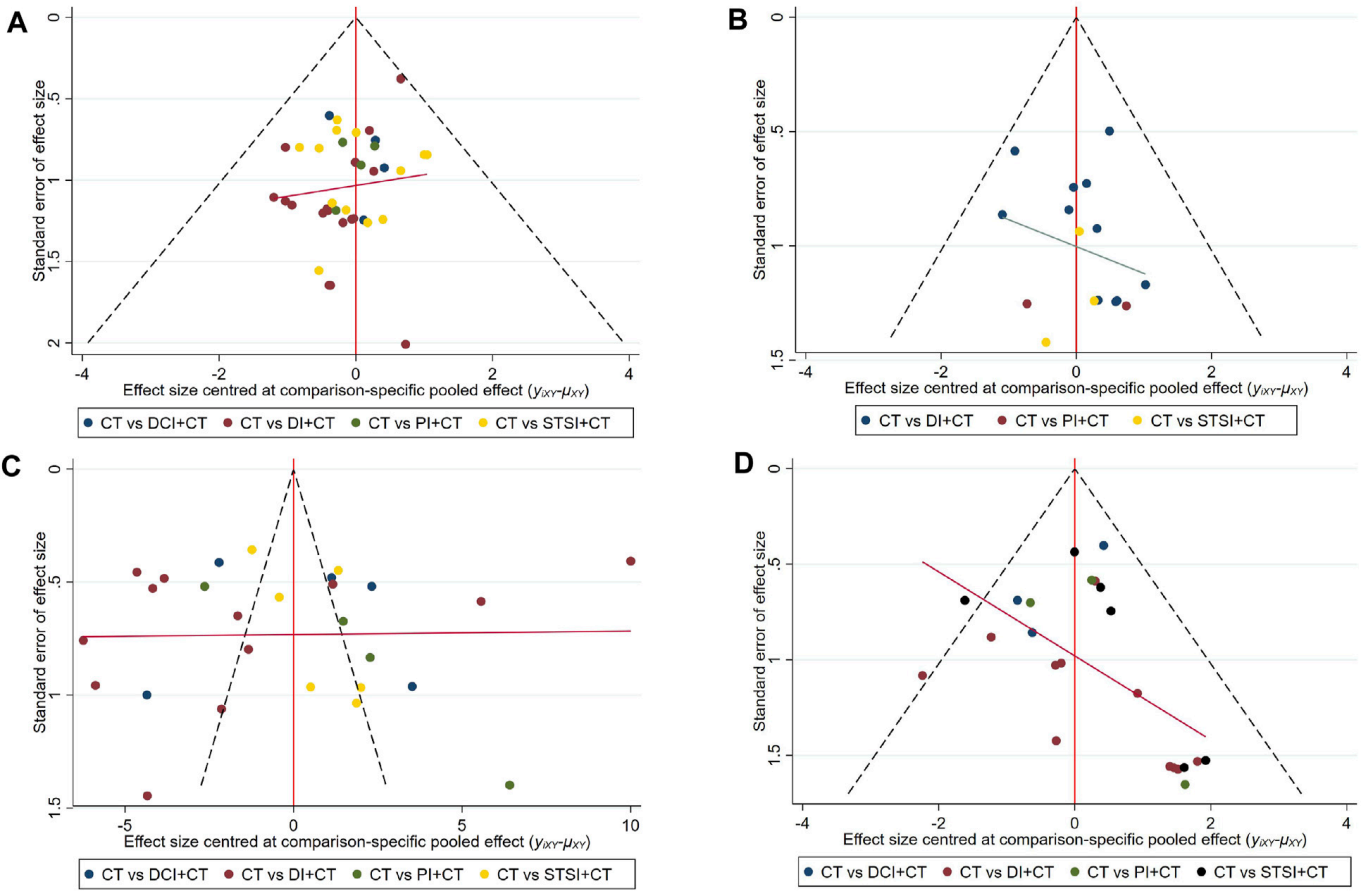
Funnel plots of the all-cause mortality, bleeding events, LVEF, and adverse events. **(A)** A funnel plot of all-cause mortality, the blue dots represent the comparison between CT and DCI + CT, the red dots represent the comparison between CT and DI + CT, the green dots represent the comparison between CT and PI + CT, the yellow dots represent the comparison between CT and STSI + CT; **(B)** a funnel plot of the incidence of bleeding events, the blue dots represent the comparison between CT and DCI + CT, the red dots represent the comparison between CT and PI + CT, the yellow dots represent the comparison between CT and STSI + CT; **(C)** a funnel plot of LVEF, the blue dots represent the comparison between CT and DCI + CT, the red dots represent the comparison between CT and DI + CT, the green dots represent the comparison between CT and PI + CT, the yellow dots represent the comparison between CT and STSI + CT; **(D)** a funnel plot of adverse events, the blue dots represent the comparison between CT and DCI + CT, the red dots represent the comparison between CT and DI + CT, the green dots represent the comparison between CT and PI + CT, the black dots represent the comparison between CT and STSI + CT. All three results show that the studies are roughly asymmetrically distributed on both sides of the X = 0 vertical line, and there is a large angle between the fitting line and the vertical line, suggesting that there may be some publication bias. “Calibration-comparison” funnel plot was created to evaluate publication bias by using Stata17.0.

Since more than 15 RCTs reported the ACM, incidence of bleeding events, LVEF, and adverse events, the publication bias of these four results was evaluated by using funnel plots (Figure 5). The dots in different colors represent the comparison between different interventions. All four results show that the studies are roughly asymmetrically distributed on both sides of the X = 0 vertical line, and there is a large angle between the fitting line and the vertical line, suggesting that there may be some publication bias. The lack of negative results and large-scale RCTs may be responsible for this bias.

### 3.7 Evidence quality evaluation

CINeMA rated the quality of evidence for each outcome as very low. Most included trials lack detailed descriptions of specific implementations of randomization and blindness in detail, so most of the within-study bias was illustrated as “some concerns.” The absence of negative results and large-scale RCTs raised “some concerns” regarding “reporting bias.” Inconsistency in baseline health conditions and reperfusion treatment measures among certain studies gives rise to “some concerns” regarding “indirectness.” The absence of a closed loop in the network limits the evaluation of inconsistency, leading to “major concerns” regarding “heterogeneity.” “Major concerns” regarding “imprecision” are present, possibly attributed to the limited number of trials available for comparison in certain outcome measures. See Supplementary Table 13 for details.

### 3.8 General meta-analysis and heterogeneity assessment

Supplementary Figures F2–F7 display forest plots of six outcomes including heterogeneity assessment. The findings revealed low heterogeneity in ACM(*I* ^2^ = 0%), bleeding events (*I* ^2^ = 0%), RMI (*I* ^2^ = 0%), and adverse events (*I* ^2^ = 22.22%). Moderate to high heterogeneity is observed in the MA (*I* ^2^ = 60.31%) and LVEF (*I* ^2^ = 98.30%). Subgroup analysis was performed based on the mean age, treatment period, sample size, reperfusion therapy type, and so on (Supplementary Tables 14, 15). The results showed that the average age of patients may be the source of heterogeneity in MA of AMI patients treated with STSI. No major causes of heterogeneity were observed in LVEF, which was assumed to be attributable to the variable levels or standards of LVEF testing between studies due to the differences in the ultrasound equipment and technical standards used for LVEF assessment.

## 4 Discussion

AMI is a deadly cardiac condition with the highest global death rates (Benjamin et al., 2019). In China, TCMI-IBCs combined with CT are commonly utilized as a therapy option for AMI intervention. TCMIs are the combination of Chinese medicine and modern technology, with characteristics such as the rapid onset of efficacy, high bioavailability, and complex composition (Jiang et al., 2016; Xiao et al., 2019; Chen et al., 2020). The flavonoid glycoside, which has been demonstrated in studies to relieve vasospasm and reduce heart rate and myocardial oxygen consumption, is the major metabolite of PI (Song et al., 1988; Zhang et al., 2013). DI is made up of Salvia miltiorrhiza (*Salvia miltiorrhiza* Bunge) and Safflower (*Carthamus tinctorius* L.), which reduces vascular resistance, acts as antioxidants, and preserves the vascular endothelium (Lyu et al., 2017; Fu Z. X. et al., 2018). Tanshinone IIA sodium sulfonate is the main metabolite of STSI, which has the efficacies of anti-lipid oxidative stress, anti-inflammation, reducing myocardial ischemia-reperfusion injury, and alleviating myocardial metabolic disorders (Li and Wang, 2011). DCI is a compound preparation of chuanxiongzine hydrochloride and danshenin that exhibits anti-atherosclerosis and blood viscosity-lowering properties (Gao et al., 2019). TCMI-IBCs mentioned above all have equivalent effects of anticoagulant and antiplatelet. (Jiang, 2011; Wang et al., 2011; Maione et al., 2014; Wei et al., 2014).

Given the Chinese society’s respect for traditional medicine and the support of medical policies, the utilization rate of TCM in general medicine hospitals throughout China had not decreased (Yu et al., 2019). However, biomedicine treatment guidelines for AMI in China have not yet recommended TCM as supplementary treatment (Gao, 2001; Cardiology and Diseases, 2010; Ren et al., 2015), and adverse events do occur on occasion as a result of clinical misuse of CCPP. As a result, it is critical to investigate the efficacy and safety of TCMI-IBCs (Practitioners, 2012; Spatz et al., 2018).

We conducted a comprehensive network meta-analysis, comparing and summarizing the effectiveness and safety of four TCMI-IBCs in treating AMI patients. The results showed that: 1) TCMI-IBCs plus CT can significantly reduce ACM and improve LVEF in AMI patients. Compared to using CT alone, there are no significant impacts on bleeding events or MA. STSI combined with CT can markedly reduce the occurrence of RMI. 2) STSI + CT had the highest probability of being the best treatment strategy to reduce ACM and RMI. DI + CT was the most likely to be the best treatment strategy to reduce MA occurrence and improve LVEF, and CT may be the most effective strategy in terms of bleeding events. 3) Among the four TCMI-IBCs, DI + CT exhibited the least favorable safety. Based on the results of all indexes, STSI can be preferred for the supplementary treatment of AMI. The quality of evidence for the above conclusions needs to be enhanced, as indicated by the findings of CINeMA. Therefore, it is recommended that clinicians make informed decisions regarding the selection of TCMI-IBCs based on individual patient conditions.

The effective metabolites of TCMIs directly enter the blood. Furthermore, because TCMIs are frequently used in conjunction with biomedicine in clinical practice, clinical safety is difficult to ensure. Fatal cases of DI and PI have reported in the past (Li, 2007; Cai et al., 2011). But retrospective studies didn’t find any correlation between the hospital mortality of AMI and the application of TCMI-IBCs (Spatz et al., 2018). Four TCMI-IBCs, when combined with CT, were demonstrated to significantly reduce ACM in AMI patients. However, most of the included studies discontinued follow-up after treatment, making long-term mortality analysis impossible to obtain. The efficacy of TCMI-IBCs on the cardiogenic mortality of AMI patients remains unknown because the included trials did not explain the cause of death. In the sensitivity analysis with the number of participants ≥80, the studies related to PI were excluded due to the small number of participants. And the result of DCI + CT *vs.* CT was different from the original result, suggesting that the number of cases could affect the accuracy of the results to some extent.

Antiplatelet and anticoagulation therapy can reduce the incidence of ischemic events in patients with AMI, but correspondingly increase the risk of bleeding (Udell et al., 2016). Balancing the risk of ischemia and bleeding is a difficult problem for clinicians. Previous studies have shown that Danshen-based TCMI can increase the risk of bleeding in AMI patients (Yu et al., 2019). In addition, when AMI patients receive routine antithrombotic therapy combined with TCMI-IBCs, more attention should be paid to lowering the bleeding risk. No significant distinction was observed between using CT alone and the combination of TCMI-IBCs with CT in terms of bleeding occurrences, according to this research. However, according to the results of SUCRA, CT was the optimal treatment strategy, suggesting that TCMI-IBCs combined with CT may increase the risk of bleeding to some extent. In clinical practice, AMI is a high-thrombosis risk event, and adequate antithrombotic therapy may be the best treatment strategy, especially for patients with relatively low risk of bleeding. Therefore, clinicians should carefully assess the thrombosis/bleeding risk of each AMI patient.

During AMI attacks, various arrhythmias, especially ventricular tachycardia and ventricular fibrillation, may occur due to myocardial ischemia and the formation of myocardial scar tissue (Zareba et al., 1994; Verma et al., 2005), which may lead to cardiac arrest in severe cases (Fang et al., 2022). Moreover, there is limited evidence of the benefit of antiarrhythmic drugs for AMI patients (Piccini et al., 2011), which poses a challenge for clinical treatment. The active metabolites of TCMIs enter the blood directly, which is complex and effecting quickly, and has the risk of arrhythmia (Wang et al., 2014). In this study, the combination of DI or STSI with CT had no effect on reducing occurrence of malignant arrhythmias in AMI patients. In view of the small number of included literatures, the improvement of MA caused by PI and DCI remains unclear and needs further study.

PCI is regarded as the therapeutic strategy to reduce the major cardiovascular adverse events and mortality after AMI (Ibanez et al., 2018), but some scholars believe that it is a risk factor for RMI (Arnold et al., 2015; Yudi et al., 2019; Song et al., 2020). PCI will damage and tear the intima of the vessel and destroy the endothelium, leading to platelet activation, and increasing the risk of acute and subacute thrombus re-occlusion and vessel restenosis after PCI (Claessen et al., 2014). Studies have shown that some patients can still develop RMI even after oral treatment with P2Y12 inhibitors (Fanaroff et al., 2018). Prevention of RMI after PCI is one of the most difficult problems in the PCI era (De Luca et al., 2021). This study found that STSI plus CT could reduce the occurrence of RMI in AMI patients, and we obtained the same conclusion in sensitivity analysis. However, given the limited number of studies included, the efficacy of PI needs further confirmation.

Myocardial ischemia and loss of functional cardiomyocytes due to coronary artery occlusion after AMI can lead to impaired pumping function of the heart (Komai et al., 2022), while reperfusion therapy can induce further myocardial injury (Hausenloy and Yellon, 2013). LVEF is an important index reflecting the contractility and number of cardiomyocytes (Park et al., 2018). In this study, it was found that TCMIs + CT had a certain efficacy in improving LVEF. In the sensitivity analysis of patients ≥80, the result of PI + CT vs. CT were different from the original result. At the same time, there is great heterogeneity in general meta-analysis, which still needs to be further explored by large-scale clinical research in the future.

In terms of adverse events, studies suggest that DI has a higher probability of adverse events, which are considered to be related to the complex composition, the great influence of solubility on the pH of the solution, and the instability of the active composition (Hu, 2019). The factors that lead to adverse events of TCMIs are complex, including excessive dose, improper compatibility, rapid drip rate, inappropriate concomitant administration and individual differences (Chen, 2013). Therefore, the supervision of the standardized use of TCMI-IBCs should be strengthened.

This study is the first systematic review using network meta-analysis to indirectly compare the efficacy and safety of four TCMI-IBCs in patients with AMI. The studies included in this meta-analysis still have the following shortcomings: 1) The methodological quality of the included studies is not high, and the generation of random sequence, allocation hiding method and blinding method are not reported in detail, which may lead to selection and implementation bias. 2) Few RCTs included in some outcomes and the existence of heterogeneity affected the reliability of results to a certain extent. 3) Baseline health status, categories of reperfusion therapy, duration of clinical application of TCMI-IBCs, definition of clinical endpoints, and duration of follow-up were inconsistent across trials, reducing the accuracy of results. 4) All the included studies were carried out in Chinese mainland, with a single ethnic group, which led to low generalization of the research conclusions. 5) There are only indirect comparisons between TCMI-IBCs, with wide confidence intervals, which may cause false negative results. 6) Treatment based on syndrome differentiation is one of the characteristics of TCM. Since most included studies did not classify participants by syndrome differentiation, the reliability of conclusions was affected. These deficiencies lead us to view the results with caution.

## 5 Conclusion

This study found that TCMI-IBCs combined with CT had potential benefits in the treatment of AMI. STSI + CT showed the best performance in the treatment of AMI, followed by DI + CT. However, patients’ specific conditions should also be considered in clinical selection of TCMI-IBCs. Due to the low quality of the original studies, additional large-scale and high-quality RCTs are needed to verified the aforementioned conclusions.

## Data Availability

The original contributions presented in the study are included in the article/Supplementary Material, further inquiries can be directed to the corresponding author.
