# Supplementary material for: Comparative effectiveness and safety of four traditional Chinese medicine injections with invigorating blood circulation, equivalent effect of anticoagulation or antiplatelet in acute myocardial infarction: a Bayesian network meta-analysis
Source: Front Pharmacol. 2024 Aug 14;15:1400990. doi: 10.3389/fphar.2024.1400990 (PMC11349691; doi:10.3389/fphar.2024.1400990)
Supplement: Supplementary file 3 [file DataSheet1.pdf]

Supplementary materials

Supplementary table S1 Composition of the four TCMI-ABCs.

TCMI-ABCs	Chinese drug name	Latin name	Botanical plant name	Family	Plant part used
Puerarin injection	Gegen	Puerariae Lobatae Radix	Pueraria montana var. lobata (Willd.) Maesen & S.M.Almeida ex Sanjappa & Predeep	Fabaceae	Root
Danhong injection	Danshen	Salviae Miltiorrhizae Radix et Rhizoma	Salvia miltiorrhiza Bunge	Lamiaceae	Rhizome and root
	Honghua	Carthami Flos	Carthamus tinctorius L.	Asteraceae	Flower
Sodium Tanshinone IIA Sulfonate injection	Danshen	Salviae Miltiorrhizae Radix et Rhizoma	Salvia miltiorrhiza Bunge	Lamiaceae	Rhizome and root
Danshen Chuanxiongqin injection	Danshen	Salviae Miltiorrhizae Radix et Rhizoma	Salvia miltiorrhiza Bunge	Lamiaceae	Rhizome and root
	Chuanxiong	Chuanxiong Rhizoma	Conioselinum anthriscoides 'Chuanxiong'	Apiaceae	Rhizome

Supplementary table S2 Initial processing, indication, adverse reactions and contraindications of the four TCMI-ABCs.

TCMI-ABCs	Description of the extract and extraction process	Indication, adverse reactions and contraindications	Source	Quality control reported. (Y/N)
Puerarin injection	The pharmaceutical factory takes the prescribed amount of puerarin and adds the appropriate amount of propylene glycol into the liquid tank. They then stirs until fully dissolved, followed by adding 90% of the prescribed amount of water for injection and stirring evenly. Next, they adjusts the pH value to about 5.0 with either a 0.1mol/L hydrochloric acid solution or a 0.1mol/L sodium hydroxide solution, and add water for injection to reach full volume. After confirming the measured content is qualified, it will be filtered through a 0.22μm microporous filter membrane, filled in ampoules, and sterilized by steam at 100℃ for 30 minutes.	Indication: The adjuvant therapy of coronary heart disease, angina pectoris, myocardial infarction, retinal arteriotomy, venous obstruction, sudden deafness.<br>Adverse reactions: 1. Some patients had temporary gastrointestinal reactions such as abdominal distension and nausea at the beginning of medication, which disappeared spontaneously with continued medication; 2. A small number of patients may appear rash, allergic asthma, anaphylactic shock, fever and other allergic reactions, very few patients appear hemolytic reaction. Once the above adverse reactions, patients should immediately stop the drug and symptomatic treatment; 3. Occasionally acute intravascular hemolysis: chills, fever, jaundice, low back pain, deepened urine color, and so on.<br>Contraindications: 1. Patients with severe liver and renal insufficiency, heart failure and other serious organic diseases are prohibited; 2. It is prohibited for those who are allergic to this medicine or allergic to it.	1. Chia Tai Tianqing Pharmaceutical Group Co., LTD<br>2. Ankang Zhengda Pharmaceutical Co., Ltd<br>3. Yantai Zhongce Pharmaceutical Co., Ltd<br>4. Hebei Changshan Biochemical Pharmaceutical Co., Ltd	Y-National Pharmaceutical Standard: H20034120
Danhong injection	The 750g salvia miltiorrhiza and 250g safflower were decocted and boiled twice with water for 1 hour each time, filtered, followed by combining filtrate, and concentrated into a clear paste with a relative density of 1.20~1.30 (65℃). Then ethanol was added to the clear paste by the pharmaceutical factory to achieve an alcohol content of 75-80%. The mixture was refrigerated, and the supernatant was taken. After recycling ethanol, the mixture was concentrated to a relative density of 1.20-1.30 (65℃) of the clear paste. Ethanol was then added	Indication: It is used for chest pain and stroke caused by blood stasis. Symptoms include chest pain, chest tightness, palpitations, mouth and eye deviation, speech impediment, limb numbness, and restricted mobility. It is also used for coronary heart disease, angina pectoris, myocardial infarction, blood stasis pulmonary heart disease, ischemic encephalopathy, and cerebral thrombosis.<br>Adverse reactions: This product may occasionally cause allergic reactions, including rash, pruritus, headache, dizziness, palpitations,	Shandong Danhong Pharmaceutical Co., LTD	Y-National Pharmaceutical Standard: Z20026866

	into mixture to make the alcohol content up to 80-85%, and the supernatant was taken. About 1% activated carbon of the mixture volume was added by the pharmaceutical factory to the upper liquid, followed by stirring for 30 minutes, being left to stand, and filtration. Ethanol was recycled from the filtrate by the pharmaceutical factory until no alcohol taste remained. Then water for injection was added to reach a volume of 1000ml. The mixture was stirred well, heated and boiled for 40-50 minutes, refrigerated and filtered. Then the mixture was concentrated to about 333ml before being refrigerated again and filtered once more. The pH value of the filtrate was adjusted with a solution containing 40% sodium hydroxide until it reached between 6.5~7.5, after which it was heated and boiled for 40~50 minutes. Adding 0.5% of the liquid volume of activated carbon, then the pharmaceutical factory let the temperature of the liquid reduced to 50~60℃, followed by stewing and filtration. After ultra filtration of the filtrate, a further 0.5% of the liquid volume of activated carbon was added before boiling and filtering. After adding water for injection to the filtrate to 1000m1, the pharmaceutical factory adjusts the pH value to 6.5~7.5, followed by filtration, sterilization, then the Danhoong injection was obtained.	chills, fever, facial flushing, nausea, vomiting, diarrhea, chest tightness, dyspnea, laryngeal edema, and convulsions. All symptoms resolve upon discontinuation of use. Rare anaphylactic shock.<br>Contraindications: 1. This product is contraindicated for individuals prone to bleeding, as well as for pregnant and lactating women; 2. Those allergic to this product should not use it.		
Sodium Tanshinone IIA Sulfonate injection	The pharmaceutical factory dries the salvia miltiorrhiza residue after boiling into coarse powder (the same process applies to raw materials not boiled). Then they weighs 50kg coarse powder, and adds five times the volume of 95% alcohol into it. The mixture is heated and refluxed for four hours, then it is filtered, followed by adding three Indication: It is used for chest pain and stroke caused by blood stasis. Symptoms include chest pain, chest tightness, palpitations, mouth and eye deviation, speech impediment, limb numbness, and restricted mobility. It is also used for coronary heart disease, angina pectoris, myocardial infarction, blood stasis pulmonary heart disease, ischemic encephalopathy, and cerebral thrombosis.<br>Adverse reactions: This product may occasionally cause allergic reactions, including rash, pruritus, headache, dizziness, palpitations, chills, fever, facial flushing, nausea, vomiting, diarrhea, chest tightness, dyspnea, laryngeal edema, and convulsions. All symptoms resolve upon discontinuation of use. Rare anaphylactic shock.<br>Contraindications: 1. This product is contraindicated for individuals prone to bleeding, as well as for pregnant and lactating women; 2. Those allergic to this product should not use it.and a half times the volume of 95% alcohol. The pharmaceutical factory heats and refluxes the mixture for three hours, followed by filtration, and repeats the process once more. The first alcohol extract is concentrated to approximately 20~25 liters, the second and third reflux extracts are combined and concentrated to the same volume, and each is left for about 15 hours for precipitation (the amount of crude product precipitated varies by variety and content), then the crude product is filtered. The mother liquor is left for about 15 hours for further precipitation, and the precipitates are filtered. The mother liquor is then concentrated to one-third of its volume, and left for over 10 hours, during which time further crude product precipitates. This	Indication: The adjuvant treatment of coronary heart disease, angina pectoris and myocardial infarction.<br>Adverse reactions: In some cases, rash, maculopapular rash, dermatitis, anaphylactic shock, chills, fever, hypotensive shock, pain, phlebitis, nausea, abdominal pain and other symptoms may occur.<br>Contraindications: People allergic to this product should not use it.	SPH NO.1 Biochemical & Pharmaceutical Co., LTD	Y-National Pharmaceutical Standard: H31022558

	process is repeated until no more precipitates are observed. Then the extract is the Sodium Tanshinone IIA Sulfonate.			
Danshen Chuanxiongqin injection	The pharmaceutical factory processes 200g Salvia miltiorrhiza, which is extracted with water and then treated with the sulfuric acid method. The extract is then refined through two alcohol precipitations (with the first reaching 60% and the second 70% alcohol content) to recycle the ethanol. This results in a clear liquid containing 0.4g of medicinal materials per 1ml, with the pH value adjusted for use as a stock solution. To prepare, mix 20g ligustrazine hydrochloride, 200ml glycerol, and the aforementioned solution thoroughly. Then the pharmaceutical factory adds water for injection and adjust the pH with hydrochloric acid solution, making a total volume of 1000ml. This mixture is then filtered, filled into 5ml ampoules, and sterilized at 115℃ for 30 minutes, then the Danshen Chuanxiongqin was obtained.	Indication: It is used for occlusive cerebrovascular diseases, such as cerebral insufficiency, cerebral thrombosis, cerebral embolism and other ischemic cardiovascular diseases, such as chest tightness of coronary heart disease, angina pectoris, myocardial infarction, ischemic stroke, thromboangiitis obliterans, and so on.<br>Adverse reactions: Patients with cerebral hemorrhage and bleeding tendency should not use it.	1. Jilin Sichang Pharmaceutical Co., Ltd<br>2. Guizhou Beite Co., Ltd	Y-National Pharmaceutical Standard: H52020959

Supplementary table S3 Analytical methods for the chemical profile of the four kinds of TCMI-ABCs according to ConPhyMP

**TCMI-ABCs**	**Type of extract**	**Preferred/main methods and component identification results for extract characterisation/chemical analysis**	**Alternative methods and component identification results for extract characterisation/chemical analysis**	**Reference**
Puerarin injection	A	Determining content using high-performance liquid chromatography<br>Octadecylsilane bonded silica gel was used as filler; The mobile phase was 0.1% citric acid solution methanol (75:25); The detection wavelength was 250nm. The number of theoretical plates shall not be less than 5000 according to puerarin peak, and the separation between puerarin peak and adjacent impurity peak shall meet the requirements.<br>Determination method: Take an appropriate amount of this product, accurately weigh it, quantitatively dilute it with mobile phase to make a solution containing about 50 μg puerarin per 1mL as the test solution. Then we accurately measure 10 μL test solution, and inject it into the liquid chromatograph to record the chromatogram; Take another puerarin as a control and determine it with the same method. Calculate the peak area according to the external standard method.	(1) Dispense an appropriate amount of the product, add 2-3 drops of a 0.5% ferric chloride solution, shake well, followed by the addition of 2-3 drops of a 0.5% potassium ferricyanide solution, shake again to mix thoroughly, and observe the resulting blue-green color.<br>(2) Dispense an appropriate amount of this product and add ethanol to prepare a solution with approximately 10 μg of puerarin per 1mL. According to the UV-spectrophotometry, there is a maximum absorption at the wavelength of 250nm.	Pharmacopoeia of the People's Republic of China

Danhong injection	A	Salvia miltiorrhiza<br>Determining content using high-performance liquid chromatography<br>Octadecylsilane bonded silica gel was used as filler, methanol-1% glacial acetic acid solution (13:87) was used as mobile phase, and the detection wavelength was 280nm. The number of theoretical plates shall not be less than 5000 according to the peak of Danshensu.<br>Preparation of reference solution: Accurately weigh an appropriate amount of Danshensu Sodium and Protocatechualdehyde reference, add water to make a solution containing 50 g per 1mL.<br>Preparation of total test solution: Precisely measure 5mL of this product, put it into a 20mL measuring flask, add water to dilute to the scale, shake well, and then get.<br>The determination method is to precisely aspirate 10mL of each of the reference and test solution, inject them into the liquid chromatograph, and determine.<br>This product contains no less than 0.5mg of Salvia miltiorrhiza per 1mL based on the total amount of Danshensu (c9h1005) and protocatechuic aldehyde (c7h603)<br>Total flavonoids<br>Preparation of reference solution: Accurately weigh 20mg of rutin reference substance dried at 120 ℃ to constant weight, put it into a 100m1 volumetric flask, add an appropriate amount of 50% methanol, shake to dissolve and dilute to the scale, and shake well to obtain (0.2mg of anhydrous rutin per 1m1).<br>Preparation of standard curve accurately measure 1.0mL, 2.0mL, 3.0mL, 4.0mL and 5.0mL of the reference solution, put them into 10mL volumetric flasks respectively, add 50% methanol to 5mL each, add 0.3mL of 5% sodium nitrite solution, shake well, place for 6 minutes, add 0.3mL of 10% aluminum nitrate solution, shake well, place for 6 minutes, add 4mL of sodium hydroxide test solution, add 50% methanol to the scale, shake well. Take the corresponding solution as blank.<br>Spectrophotometric method: Determine the absorbance at the wavelength of	Evaporate 4mL of this product to dryness, add 1mL of absolute ethanol to the residue, dissolve, centrifuge, and use the supernatant as the test solution. Additionally, take 1g of Carthamus tinctorius control medicinal material, add 10mL of water, sonicate for 30 minutes, filter, concentrate the filtrate to dryness, add 1mL of absolute ethanol to the residue, dissolve, centrifuge, and use the supernatant as the control medicinal material solution. Conduct a thin-layer chromatography test by applying 1μL of each solution to the same silica gel G plate, using n-butanol-glacial acetic acid-water (6:2.4:5) as the developer, develop, remove, dry, and inspect under ultraviolet light (365nm). In the chromatogram of the test sample, fluorescent spots of the same color appear at the position corresponding to the chromatogram of the control medicinal material.	National Drug Standards of China Food and Drug Administration (WS-11220（ZD-1220）-2002)

		500nm, and draw a standard curve with the absorbance as the ordinate and the concentration as the abscissa.<br>Determination method: Accurately suck 5mL of this product, place it in a 100mL measuring flask, add water to dilute to the scale, shake well, accurately measure 1mL, place it in a 10mL measuring flask, add 50% methanol to the scale, shake well, and serve as a blank control. In addition, accurately measure 1mL, put it into a 10mL measuring flask, measure the absorbance according to the method under the preparation of the standard curve, read the weight equivalent to Rutin in the test solution from the standard curve, and calculate.<br>The total flavonoids contained in this product per 1mL shall not be less than 5.0mg based on rutin (c27h30016).		
Sodium Tanshinone IIA Sulfonate injection	A	Take about 25mg of the product, accurately weigh it, put it into a 250ml measuring bottle, add water to dilute to the scale, shake it well, accurately measure 5ml, put it into a 100ml measuring bottle, add water to dilute to the scale, shake it well, use spectrophotometry, measure the absorbance at the wavelength of (271 ± 2) nm, and calculate according to the absorption coefficient of $C_{19}H_{17}O_3 \cdot SO_3Na$ is 767.	⑴ Take approximately 0.5mg of this substance, place it on a white porcelain plate, add 1 to 2 drops of sulfuric acid. It immediately turns dark blue, gradually changing to dark green.<br>⑵ Take 10mg of the substance, ignite to ash, add 5mL of 1mol/L hydrochloric acid solution, filter the mixture, then add 5mL of barium oxide test solution to the filtrate. This results in a white precipitate.<br>⑶ Take the solution prepared for the content determination, and measure it using spectrophotometry. It exhibits maximum absorption at wavelengths of (226 ± 2) nm, (253 ± 2) nm, and (271 ± 2) nm. Additionally, it displays a characteristic peak at a wavelength of (280 ± 2) nm.	National Drug Standards of China Food and Drug Administration (WS-10001-(HD-1014)-2002)
Danshen Chuanxiongqin injection	A	Danshensu<br>Determining content using high-performance liquid chromatography<br>Octadecane bonded silica gel was used as filler; The mobile phase was methanol-0.2% glacial acetic acid (15：85), the flow rate was 1.0mL/min, the detection wavelength was 280nm, and the number of theoretical plates	(1) Take 1ml of this product and add 2-3 drops of ferric chloride test solution to make it dirty green.<br>(2) In the chromatogram under the content determination item, the main peak of the test solution should be consistent with the peak retention time of the control solution.	National Drug Standards of China Food and Drug Administration (WS-10001-(HD-1138)-

		should not be less than 1500 according to the tanshinol peak.<br>Preparation of reference solution take an appropriate amount of Danshensu Sodium reference, accurately weigh, add methanol to dissolve and quantitatively dilute to make a solution containing about 90mg per 1mL (1mg of Danshensu Sodium is equivalent to 0.875mg of Danshensu).<br>Preparation of test solution accurately measure 5~25mL of this product, place it in a volumetric flask, add methanol to dilute to the scale, shake well, filter with a microporous filter membrane (0.45mm), and take the filtrate as the test solution.<br>Determination method take 10mL of the reference solution and 10mL of the test solution respectively, inject them into the chromatograph, record the chromatogram, and calculate the peak area according to the external standard method.<br><br>Ligustrazine hydrochloride<br>Determining content using high-performance liquid chromatography<br>Octadecylsilane bonded silica gel was used as filler; Methanol-0.4% phosphoric acid (10：90) was used as the mobile phase, the flow rate was 1.0mL/min, the detection wavelength was 292nm, and the number of theoretical plates according to the peak of ligustrazine hydrochloride should not be less than 2000.<br>Preparation of reference solution: Take an appropriate amount of tetramethylpyrazine hydrochloride standard, accurately weigh it, dissolve it with methanol and quantitatively dilute it to make a solution containing about 40mg per 1mL.<br>Preparation of test solution: Accurately measure 1mL of this product into a 50mL volumetric flask, add methanol to dilute to the scale, shake, take 1mL of diluted solution into a 10mL volumetric flask, add methanol to dilute to the scale, shake, filter with a microporous filter membrane (0.45mm), take the filtrate as the test solution.<br>For the determination method, 5mL of the reference solution and 5mL of the		2002)

		test solution were accurately aspirated, injected into the chromatograph, and the chromatogram was recorded. The peak area was calculated according to the external standard method.		

Supplementary table S4 The principal chemical constituents of four TCMI-ABCs under fingerprint methods.

**TCMI-ABCs**	**Literature sources**	**fingerprinting methods**	**chemical constituents**
Puerarin injection	(Li et al., 2014)	HPLC	Puerarin, flavonoid glycoside
Danhong injection	(Wang et al., 2014)	HPLC-UV-MS	5-hydroxymethylfurfural, Sodium Danshensu, Protamine sulfates, 2-Oxo-2H-pyran-5-carboxylic acid, Salvianolic acid D, Rosmarinic acid, salvianolic acid B, Salvianolic acid A, Nicotiflorin
	(Li et al., 2022)	^{1}H-qNMR	tert-Leucine, l-isoleucine, Valine, DL-Threonine, L-Alanine, L-glutamic acid, DL-Glutamine, Proline, ANION STANDARD-SUCCINATE, ethyl 5-amino-6-iodopyridine-2-carboxylate, Fructose, sucrose, D-Galactose, Xylosucrose, 5-hydroxymethylfurfural, Danshensu, Protamine sulfates, Rosmarinic acid, Lithospermic acid, salvianolic acid B, Salvianolic acid A, Salvianolic acid D, 2-Oxo-2H-pyran-5-carboxylic acid
Sodium Tanshinone IIA Sulfonate injection	(Ding et al., 2007)	HPLC	Tanshinone IIA sodium sulfonate
Danshen Chuanxiongqin injectio	(Zhou et al., 2019)	UHPLC-Q-Orbitrap HRMS	Tetramethylpyrazine, Danshensu, Salvianolic acid A, Succinic acid, Rosmarinic acid, Protamine sulfates, Caffeic acid
	(Li et al., 2008)	HPLC	Tanshinone IIA, Tetramethylpyrazine

References

1. Li C Q, Li D, Wang P Q, et al. Determination of puerarin content in 3 puerarin injections by hplc. Journal of Chinese Experimental Formulae. 2014, 20 (24): 60-63.
2. Wang Y, Shao Q, Ju H B, et al. Study on multi-fingerprint and multi-component quantitative analysis of danhong injection. Chinese Traditional and Herbal Drugs. 2014, 45 (04): 490-497.
3. Li W Z, Xie X Y, Yang J Y, et al. Nmr method for simultaneous determination of 21 chemical components in danhong injection. Chinese Journal of Traditional Chinese Medicine. 2022, 47 (23): 6399-6408.
4. Ding X J. Preparation and related substances of sodium tanshinone ii a sulfonate reference substance. Tianjin University, 2007: 76.
5. Zhou P P, Zhou L, Sun Z, et al. Identification and content determination of main chemical components of salvia miltiorrhiza ligustrazine injection based on uhplc-q-orbitrap hrms technology. Chinese Journal of Pharmacy. 2019, 54 (04): 327-333.
6. Li W, Zhong S H, Guo L M, et al. Determination of tanshinone ii a and ligustrazine in salvia miltiorrhiza and ligustrazine injection by hplc. China Medical Herald. 2008, (34): 13-15.

Supplementary table S5 The details of search terms and literature search strategy

Take searching PubMed as an example, the search terms and strategies are as follows:

Search	Query
#1	Myocardial Infarction[MeSH]
#2	Infarction, Myocardial[TIAB]
#3	Infarctions, Myocardial[TIAB]
#4	Myocardial Infarctions[TIAB]
#5	Cardiovascular Stroke[TIAB]
#6	Cardiovascular Strokes[TIAB]
#7	Stroke, Cardiovascular[TIAB]
#8	Strokes, Cardiovascular[TIAB]
#9	Myocardial Infarct[TIAB]
#10	Infarct, Myocardial[TIAB]
#11	Infarcts, Myocardial[TIAB]
#12	Myocardial Infarcts[TIAB]
#13	Heart Attack[TIAB]
#14	Heart Attacks[TIAB]
#15	Inferior Wall Myocardial Infarction[TIAB]
#16	Diaphragmatic Myocardial Infarction[TIAB]
#17	Diaphragmatic Myocardial Infarctions[TIAB]
#18	Infarction, Diaphragmatic Myocardial[TIAB]
#19	Infarctions, Diaphragmatic Myocardial[TIAB]
#20	Myocardial Infarction, Diaphragmatic[TIAB]
#21	Myocardial Infarctions, Diaphragmatic[TIAB]
#22	Myocardial Infarction, Inferior Wall[TIAB]
#23	Inferior Myocardial Infarction[TIAB]
#24	Infarction, Inferior Myocardial[TIAB]
#25	Infarctions, Inferior Myocardial[TIAB]
#26	Inferior Myocardial Infarctions[TIAB]
#27	Myocardial Infarction, Inferior[TIAB]
#28	Myocardial Infarctions, Inferior[TIAB]

#29	Acute Inferior Myocardial Infarction[TIAB]
#30	Anterior Wall Myocardial Infarction[TIAB]
#31	Myocardial Infarction, Anterior Wall[TIAB]
#32	Anterolateral Myocardial Infarction[TIAB]
#33	Anterolateral Myocardial Infarctions[TIAB]
#34	Infarction, Anterolateral Myocardial[TIAB]
#35	Infarctions, Anterolateral Myocardial[TIAB]
#36	Myocardial Infarction, Anterolateral[TIAB]
#37	Myocardial Infarctions, Anterolateral[TIAB]
#38	Anteroseptal Myocardial Infarction[TIAB]
#39	Anteroseptal Myocardial Infarctions[TIAB]
#40	Infarction, Anteroseptal Myocardial[TIAB]
#41	Infarctions, Anteroseptal Myocardial[TIAB]
#42	Myocardial Infarction, Anteroseptal[TIAB]
#43	Myocardial Infarctions, Anteroseptal[TIAB]
#44	Acute Anterior Wall Myocardial Infarction[TIAB]
#45	Non-ST Elevated Myocardial Infarction[TIAB]
#46	Non ST Elevated Myocardial Infarction[TIAB]
#47	NSTEMI[TIAB]
#48	Non-ST-Elevation Myocardial Infarction[TIAB]
#49	Infarction, Non-ST-Elevation Myocardial[TIAB]
#50	Infarctions, Non-ST-Elevation Myocardial[TIAB]
#51	Myocardial Infarction, Non-ST-Elevation[TIAB]
#52	Myocardial Infarctions, Non-ST-Elevation[TIAB]
#53	Non ST Elevation Myocardial Infarction[TIAB]
#54	Non-ST-Elevation Myocardial Infarctions[TIAB]
#55	ST Elevation Myocardial Infarction[TIAB]
#56	ST Segment Elevation Myocardial Infarction[TIAB]
#57	ST Elevated Myocardial Infarction[TIAB]
#58	STEMI[TIAB]
#59	#1-58/OR

#60	Puerarin injection[TIAB]
#61	Puerarin[TIAB]
#62	#60-61/OR
#63	Danhong Injection[TIAB]
#64	Dan red injection[TIAB]
#65	#63-64/OR
#66	Sulfotanshinone sodium injection[TIAB]
#67	Sodium Tanshinone IIA Sulfonate[TIAB]
#68	Tanshinone IIa injection[TIAB]
#69	#66-68/OR
#70	Salviae miltiorrhizae and ligustrazine hydrochloride injection[TIAB]
#71	Danshen Chuanxiongqin Injection[TIAB]
#72	Clinical Trial[Publication Type]
#73	#70-71/OR
#74	#59 AND #62 AND #72
#75	#59 AND #65 AND #72
#76	#59 AND #69 AND #72
#77	#59 AND #72 AND #72
#78	#74 0R #75 0R #76 0R #77

Supplementary table S6 The details of TCMIs of all the included studies

Study	Formulation	Source	Quality control reported? (Y/N)	Chemial analysis reported? (Y/N)
Li, et al(2002)	Puerarin injection	-	Y-Prepared according to pharmacopoeia of the People's Republic of China	N
Gu, et al(2004)	Puerarin injection	Chia Tai Tianqing Pharmaceutical Group Co., LTD	Y-Prepared according to pharmacopoeia of the People's Republic of China	N
Qu, et al(2008)	Puerarin injection	Ankang Zhengda Pharmaceutical Co., Ltd	Y-Prepared according to pharmacopoeia of the People's Republic of China	N
Xiao, et al(2004)	Puerarin injection	Ankang Zhengda Pharmaceutical Co., Ltd	Y-Prepared according to pharmacopoeia of the People's Republic of China	N
Lv, et al(2001)	Puerarin injection	Yantai Zhongce Pharmaceutical Co., Ltd	Y-Prepared according to pharmacopoeia of the People's Republic of China	N
Dong, et al(2022)	Puerarin injection	-	Y-Prepared according to pharmacopoeia of the People's Republic of China	N
Liu, et al(2008)	Puerarin injection	-	Y-Prepared according to pharmacopoeia of the People's Republic of China	N
Fan, et al(2017)	Puerarin injection	Hebei Changshan Biochemical Pharmaceutical Co., Ltd	Y-Prepared according to pharmacopoeia of the People's Republic of China	N
Hu, et al(2018)	Danhong injection	Shandong Danhong Pharmaceutical Co., Ltd	Y-Prepared according to National Drug Standards of China Food and Drug Administration (WS-11220（ZD-1220）-2002)	N
Chen, et al(2021)	Danhong injection	Shandong Danhong Pharmaceutical Co., Ltd	Y-Prepared according to National Drug Standards of China Food and Drug Administration (WS-11220（ZD-1220）-2002)	N
Huo, et al(2019)	Danhong injection	-	Y-Prepared according to National Drug Standards of China Food and Drug Administration (WS-11220（ZD-1220）-2002)	N
Yang, et al(2015)	Danhong injection	-	Y-Prepared according to National Drug Standards of China Food and Drug Administration (WS-11220（ZD-1220）-2002)	N
Jin, et al(2019)	Danhong injection	Shandong Buchang Pharmaceutical Co., Ltd	Y-Prepared according to National Drug Standards of China Food and Drug Administration (WS-11220（ZD-1220）-2002)	N
You, et al(2019)	Danhong injection	Shandong Danhong Pharmaceutical Co., Ltd	Y-Prepared according to National Drug Standards of China Food and Drug Administration (WS-11220（ZD-1220）-2002)	N
Ji, et al(2016)	Danhong injection	-	Y-Prepared according to National Drug Standards of China Food and Drug Administration (WS-11220（ZD-1220）-	N

			2002)	
Qin, et al(2014)	Danhong injection	Shandong Heze Buchang Pharmaceutical Co., Ltd	Y-Prepared according to National Drug Standards of China Food and Drug Administration (WS-11220（ZD-1220）-2002)	N
Ren, et al(2014)	Danhong injection	Jinan Buchang Pharmaceutical Co., Ltd	Y-Prepared according to National Drug Standards of China Food and Drug Administration (WS-11220（ZD-1220）-2002)	N
Feng, et al(2021)	Danhong injection	-	Y-Prepared according to National Drug Standards of China Food and Drug Administration (WS-11220（ZD-1220）-2002)	N
Li, et al(2020)	Danhong injection	Shandong Danhong Pharmaceutical Co., Ltd	Y-Prepared according to National Drug Standards of China Food and Drug Administration (WS-11220（ZD-1220）-2002)	N
Chen LF, et al(2021)	Danhong injection	Shandong Danhong Pharmaceutical Co., Ltd	Y-Prepared according to National Drug Standards of China Food and Drug Administration (WS-11220（ZD-1220）-2002)	N
Hao, et al(2011)	Danhong injection	Jinan Buchang Pharmaceutical Co., Ltd	Y-Prepared according to National Drug Standards of China Food and Drug Administration (WS-11220（ZD-1220）-2002)	N
Zhang, et al(2021)	Danhong injection	Shandong Danhong Pharmaceutical Co., Ltd	Y-Prepared according to National Drug Standards of China Food and Drug Administration (WS-11220（ZD-1220）-2002)	N
Zhang, et al(2012)	Danhong injection	Shanghai Hehuang Pharmaceutical Co., Ltd	Y-Prepared according to National Drug Standards of China Food and Drug Administration (WS-11220（ZD-1220）-2002)	N
Zhang, et al(2019)	Danhong injection	-	Y-Prepared according to National Drug Standards of China Food and Drug Administration (WS-11220（ZD-1220）-2002)	N
Gao, et al(2009)	Danhong injection	-	Y-Prepared according to National Drug Standards of China Food and Drug Administration (WS-11220（ZD-1220）-2002)	N
Li, et al(2019)	Danhong injection	Shandong Danhong Pharmaceutical Co., Ltd	Y-Prepared according to National Drug Standards of China Food and Drug Administration (WS-11220（ZD-1220）-2002)	N
Liu, et al(2015)	Danhong injection	Shandong Heze Buchang Pharmaceutical Co., Ltd	Y-Prepared according to National Drug Standards of China Food and Drug Administration (WS-11220（ZD-1220）-2002)	N
Zhang, et al(2018)	Danhong injection	Shandong Danhong Pharmaceutical Co., Ltd	Y-Prepared according to National Drug Standards of China Food and Drug Administration (WS-11220（ZD-1220）-2002)	N
Gui, et al(2009)	Danhong injection	-	Y-Prepared according to National Drug Standards of China Food and Drug Administration (WS-11220（ZD-1220）-2002)	N
Chen, et al(2010)	Danhong injection	Shandong Heze Buchang Pharmaceutical Co., Ltd	Y-Prepared according to National Drug Standards of China Food and Drug Administration (WS-11220（ZD-1220）-2002)	N
Han, et al(2012	Danhong injection	-	Y-Prepared according to National Drug Standards of China Food and Drug	N

)			Administration (WS-11220（ZD-1220）-2002)	
Han, et al(2010)	Danhong injection	-	Y-Prepared according to National Drug Standards of China Food and Drug Administration (WS-11220（ZD-1220）-2002)	N
Xu, et al(2015)	Danhong injection	Shandong Danhong Pharmaceutical Co., Ltd	Y-Prepared according to National Drug Standards of China Food and Drug Administration (WS-11220（ZD-1220）-2002)	N
Cui, et al(2021)	Danhong injection	Shandong Danhong Pharmaceutical Co., Ltd	Y-Prepared according to National Drug Standards of China Food and Drug Administration (WS-11220（ZD-1220）-2002)	N
Wang, et al(2015)	Danhong injection	Shandong Danhong Pharmaceutical Co., Ltd	Y-Prepared according to National Drug Standards of China Food and Drug Administration (WS-11220（ZD-1220）-2002)	N
Zhao, et al(2012)	Danhong injection	Jinan Buchang Pharmaceutical Co., Ltd	Y-Prepared according to National Drug Standards of China Food and Drug Administration (WS-11220（ZD-1220）-2002)	N
Zeng, et al(2017)	Danhong injection	Jinan Buchang Pharmaceutical Co., Ltd	Y-Prepared according to National Drug Standards of China Food and Drug Administration (WS-11220（ZD-1220）-2002)	N
You Q, et al(2019)	Danhong injection	Shandong Danhong Pharmaceutical Co., Ltd	Y-Prepared according to National Drug Standards of China Food and Drug Administration (WS-11220（ZD-1220）-2002)	N
Fu, et al(2018)	Danhong injection	Shandong Danhong Pharmaceutical Co., Ltd	Y-Prepared according to National Drug Standards of China Food and Drug Administration (WS-11220（ZD-1220）-2002)	N
Zhou, et al(2010)	Danhong injection	Jinan Buchang Pharmaceutical Co., Ltd	Y-Prepared according to National Drug Standards of China Food and Drug Administration (WS-11220（ZD-1220）-2002)	N
Chen, et al(2013)	Danhong injection	Jinan Buchang Pharmaceutical Co., Ltd	Y-Prepared according to National Drug Standards of China Food and Drug Administration (WS-11220（ZD-1220）-2002)	N
Shen, et al(2019)	Danhong injection	Shandong Heze Buchang Pharmaceutical Co., Ltd	Y-Prepared according to National Drug Standards of China Food and Drug Administration (WS-11220（ZD-1220）-2002)	N
Liu, et al(2014)	Sodium Tanshinone IIA Sulfonate injection	-	Y-Prepared according to National Drug Standards of China Food and Drug Administration (WS-10001-(HD-1014)-2002)	N
Qi, et al(2006)	Sodium Tanshinone IIA Sulfonate injection	Barrymore Pharmaceutical (Tonghua) Co., Ltd	Y-Prepared according to National Drug Standards of China Food and Drug Administration (WS-10001-(HD-1014)-2002)	N
Luo RX, et al(2018)	Sodium Tanshinone IIA Sulfonate injection	Shanghai First Biochemical Pharmaceutical Co., Ltd	Y-Prepared according to National Drug Standards of China Food and Drug Administration (WS-10001-(HD-1014)-2002)	N
Chen, et al(2007)	Sodium Tanshinone IIA Sulfonate injection	Shanghai First Biochemical Pharmaceutical Co., Ltd	Y-Prepared according to National Drug Standards of China Food and Drug Administration (WS-10001-(HD-1014)-2002)	N
Qin, et al(2020	Sodium Tanshinone IIA	Shanghai First Biochemical	Y-Prepared according to National Drug Standards of China Food and Drug	N

)	Sulfonate injection	Pharmaceutical Co., Ltd	Administration (WS-10001-(HD-1014)-2002)	
Kong, et al(2013)	Sodium Tanshinone IIA Sulfonate injection	Shanghai First Biochemical Pharmaceutical Co., Ltd	Y-Prepared according to National Drug Standards of China Food and Drug Administration (WS-10001-(HD-1014)-2002)	N
Du, et al(2011)	Sodium Tanshinone IIA Sulfonate injection	-	Y-Prepared according to National Drug Standards of China Food and Drug Administration (WS-10001-(HD-1014)-2002)	N
Jia, et al(2010)	Sodium Tanshinone IIA Sulfonate injection	Hapharm Group Co., Ltd	Y-Prepared according to National Drug Standards of China Food and Drug Administration (WS-10001-(HD-1014)-2002)	N
Feng, et al(2012)	Sodium Tanshinone IIA Sulfonate injection	Shanghai First Biochemical Pharmaceutical Co., Ltd	Y-Prepared according to National Drug Standards of China Food and Drug Administration (WS-10001-(HD-1014)-2002)	N
Liu, et al(2016)	Sodium Tanshinone IIA Sulfonate injection	Shanghai First Biochemical Pharmaceutical Co., Ltd	Y-Prepared according to National Drug Standards of China Food and Drug Administration (WS-10001-(HD-1014)-2002)	N
Sun, et al(2011)	Sodium Tanshinone IIA Sulfonate injection	Shanghai First Biochemical Pharmaceutical Co., Ltd	Y-Prepared according to National Drug Standards of China Food and Drug Administration (WS-10001-(HD-1014)-2002)	N
Yu, et al(2008)	Sodium Tanshinone IIA Sulfonate injection	Barrymore Pharmaceutical (Tonghua) Co., Ltd	Y-Prepared according to National Drug Standards of China Food and Drug Administration (WS-10001-(HD-1014)-2002)	N
Fan, et al(2015)	Sodium Tanshinone IIA Sulfonate injection	Shanghai First Biochemical Pharmaceutical Co., Ltd	Y-Prepared according to National Drug Standards of China Food and Drug Administration (WS-10001-(HD-1014)-2002)	N
Song, et al(2022)	Sodium Tanshinone IIA Sulfonate injection	Shanghai First Biochemical Pharmaceutical Co., Ltd	Y-Prepared according to National Drug Standards of China Food and Drug Administration (WS-10001-(HD-1014)-2002)	N
Cao, et al(2019)	Sodium Tanshinone IIA Sulfonate injection	Shanghai First Biochemical Pharmaceutical Co., Ltd	Y-Prepared according to National Drug Standards of China Food and Drug Administration (WS-10001-(HD-1014)-2002)	N
Luo, et al(2018)	Sodium Tanshinone IIA Sulfonate injection	Shanghai First Biochemical Pharmaceutical Co., Ltd	Y-Prepared according to National Drug Standards of China Food and Drug Administration (WS-10001-(HD-1014)-2002)	N
Zhang, et al(2017)	Sodium Tanshinone IIA Sulfonate injection	Shanghai First Biochemical Pharmaceutical Co., Ltd	Y-Prepared according to National Drug Standards of China Food and Drug Administration (WS-10001-(HD-1014)-2002)	N
Lu, et al(2021)	Sodium Tanshinone IIA Sulfonate injection	Shanghai First Biochemical Pharmaceutical Co., Ltd	Y-Prepared according to National Drug Standards of China Food and Drug Administration (WS-10001-(HD-1014)-2002)	N
Mao, et al(2019)	Sodium Tanshinone IIA Sulfonate injection	-	Y-Prepared according to National Drug Standards of China Food and Drug Administration (WS-10001-(HD-1014)-2002)	N
Huang, et al(2012)	Sodium Tanshinone IIA Sulfonate injection	Shanghai First Biochemical Pharmaceutical Co., Ltd	Y-Prepared according to National Drug Standards of China Food and Drug Administration (WS-10001-(HD-1014)-2002)	N
Gao, et al(2015)	Sodium Tanshinone IIA Sulfonate	Shanghai First Biochemical Pharmaceutical Co.,	Y-Prepared according to National Drug Standards of China Food and Drug Administration (WS-10001-(HD-1014)-	N

	injection	Ltd	2002)	
Zhou, et al(2018)	Danshen Chuanxiongqin injection	Guizhou Beite Co., Ltd	Y-Prepared according to National Drug Standards of China Food and Drug Administration (WS-10001-(HD-1138)-2002)	N
Sun, et al(2016)	Danshen Chuanxiongqin injection	Jilin Sichang Pharmaceutical Co., Ltd	Y-Prepared according to National Drug Standards of China Food and Drug Administration (WS-10001-(HD-1138)-2002)	N
Liu, et al(2020)	Danshen Chuanxiongqin injection	Guizhou Beite Co., Ltd	Y-Prepared according to National Drug Standards of China Food and Drug Administration (WS-10001-(HD-1138)-2002)	N
Liang, et al(2015)	Danshen Chuanxiongqin injection	Guizhou Beite Co., Ltd	Y-Prepared according to National Drug Standards of China Food and Drug Administration (WS-10001-(HD-1138)-2002)	N
Huang, et al(2016)	Danshen Chuanxiongqin injection	Jilin Sichang Pharmaceutical Co., Ltd	Y-Prepared according to National Drug Standards of China Food and Drug Administration (WS-10001-(HD-1138)-2002)	N
Zhou, et al(2014)	Danshen Chuanxiongqin injection	Guizhou Beite Co., Ltd	Y-Prepared according to National Drug Standards of China Food and Drug Administration (WS-10001-(HD-1138)-2002)	N
Xu, et al(2017)	Danshen Chuanxiongqin injection	Guizhou Beite Co., Ltd	Y-Prepared according to National Drug Standards of China Food and Drug Administration (WS-10001-(HD-1138)-2002)	N
Zhu, et al(2020)	Danshen Chuanxiongqin injection	Guizhou Beite Co., Ltd	Y-Prepared according to National Drug Standards of China Food and Drug Administration (WS-10001-(HD-1138)-2002)	N
Miao, et al(2018)	Danshen Chuanxiongqin injection	Guizhou Beite Co., Ltd	Y-Prepared according to National Drug Standards of China Food and Drug Administration (WS-10001-(HD-1138)-2002)	N
Zhou, et al(2017)	Danshen Chuanxiongqin injection	-	Y-Prepared according to National Drug Standards of China Food and Drug Administration (WS-10001-(HD-1138)-2002)	N

Supplementary figure F1 Summary of results from assessment of studies using the Cochrane risk of bias tool.

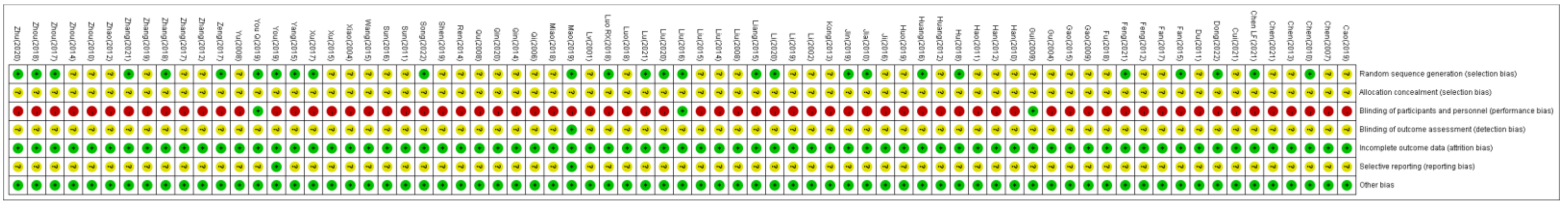

Supplementary table S7 Bayesian ranking results of network meta-analysis (all-cause mortality). The number in each cell represents the posterior probability of the row-defining treatment being ranked at the column-defining position.

**Treatments**	**Rank of possibility %**				
	**1**	**2**	**3**	**4**	**5**
Puerarin Injection	27	19	22	30	2
Danhong injection	11	27	38	24	0
Sodium Tanshinone IIA Sulfonate injection	44	34	17	6	0
Danshen Chuanxiongqin injection	19	20	24	36	2
CT	0	0	0	4	96

Supplementary table S8 Bayesian ranking results of network meta-analysis (Incidence rate of bleeding events). The number in each cell represents the posterior probability of the row-defining treatment being ranked at the column-defining position.

**Treatments**	**Rank of possibility %**			
	**1**	**2**	**3**	**4**
Puerarin Injection	23	44	29	4
Danhong injection	14	31	36	18
Sodium Tanshinone IIA Sulfonate injection	18	12	19	51
CT	45	13	16	26

Supplementary table S9 Bayesian ranking results of network meta-analysis (malignant arrhythmias). The number in each cell represents the posterior probability of the row-defining treatment being ranked at the column-defining position.

**Treatments**	**Rank of possibility %**		
	**1**	**2**	**3**
Danhong injection	63	28	9
Sodium Tanshinone IIA Sulfonate injection	37	55	8
CT	1	17	83

Supplementary table S10 Bayesian ranking results of network meta-analysis (recurrent myocardial infarction). The number in each cell represents the posterior probability of the row-defining treatment being ranked at the column-defining position.

Treatments	Rank of possibility %			
	1	2	3	4
Danhong injection	11	40	39	10
Sodium Tanshinone IIA Sulfonate injection	78	17	5	1
Danshen Chuanxiongqin injection	11	42	38	8
CT	0	1	18	81

Supplementary table S11 Bayesian ranking results of network meta-analysis (LVEF). The number in each cell represents the posterior probability of the row-defining treatment being ranked at the column-defining position.

Treatments	Rank of possibility %				
	1	2	3	4	5
Puerarin Injection	27	28	25	19	0
Danhong injection	53	32	12	3	0
Sodium Tanshinone IIA Sulfonate injection	12	24	35	29	0
Danshen Chuanxiongqin injection	7	16	29	47	1
CT	0	0	0	2	98

Supplementary table S12 Bayesian ranking results of network meta-analysis (Adverse events). The number in each cell represents the posterior probability of the row-defining treatment being ranked at the column-defining position.

Treatments	Rank of possibility %				
	1	2	3	4	5
Puerarin Injection	42	28	11	10	8
Danhong injection	2	7	13	29	50
Sodium Tanshinone IIA Sulfonate injection	4	13	19	30	34
Danshen Chuanxiongqin injection	48	31	10	7	4
CT	4	22	48	24	3

Supplementary table S13 CINeMA results of network meta-analysis.

Comparison	N.	Within-study bias	Reporting bias	Indirectness	Imprecision	Heterogeneity	Incoherence	Confidence rating
all-cause mortality	Mixed evidence							
PI:CT	4	Some concerns	No concerns	No concerns	No concerns	Major concerns	Major concerns	Very low
DI:CT	17	Some concerns	Some concerns	Some concerns	No concerns	No concerns	Major concerns	Very low
STSI:CT	13	Some concerns	Some concerns	Some concerns	No concerns	No concerns	Major concerns	Very low
DCI:CT	4	Some concerns	No concerns	No concerns	No concerns	No concerns	Major concerns	Very low
all-cause mortality	Indirect evidence							
PI:DI	0	Some concerns	Some concerns	Some concerns	Major concerns	No concerns	Major concerns	Very low
PI:STSI	0	Some concerns	Some concerns	Some concerns	Major concerns	No concerns	Major concerns	Very low
PI:DCI	0	Some concerns	No concerns	No concerns	Major concerns	No concerns	Major concerns	Very low
DI:STSI	0	Some concerns	Some concerns	Some concerns	Major concerns	No concerns	Major concerns	Very low
DI:DCI	0	Some concerns	Some concerns	Some concerns	Major concerns	No concerns	Major concerns	Very low
STSI:DCI	0	Some concerns	Some concerns	Some concerns	Major concerns	No concerns	Major concerns	Very low
Incidence of bleeding events	Mixed evidence							
PI:CT	2	Some concerns	No concerns	No concerns	Major concerns	No concerns	Major concerns	Very low
DI:CT	11	Some concerns	Some concerns	Some concerns	Major concerns	No concerns	Major concerns	Very low
STSI:CT	3	Some concerns	No concerns	No concerns	Major concerns	No concerns	Major concerns	Very low
Incidence of bleeding events	Indirect evidence							
PI:DI	0	Some concerns	Some concerns	Some concerns	Major concerns	No concerns	Major concerns	Very low
PI:STSI	0	Some concerns	No concerns	No concerns	Major concerns	No concerns	Major concerns	Very low
DI:STSI	0	Some concerns	Some concerns	Some concerns	Major concerns	No concerns	Major concerns	Very low
malignant arrhythmias	Mixed evidence							
DI:CT	4	Some concerns	No concerns	Some concerns	Major concerns	No concerns	Major concerns	Very low
STSI:CT	6	Some concerns	Some concerns	No concerns	Major concerns	No concerns	Major concerns	Very low

malignant arrhythmias	Indirect evidence							
DI:STSI	0	Some concerns	Some concerns	Some concerns	Major concerns	No concerns	Major concerns	Very low
recurrent myocardial infarction	Mixed evidence							
DI:CT	6	Some concerns	Some concerns	Some concerns	Major concerns	No concerns	Major concerns	Very low
STSI:CT	3	Some concerns	No concerns	Some concerns	No concerns	No concerns	Major concerns	Very low
DCI:CT	4	Some concerns	No concerns	Some concerns	Major concerns	No concerns	Major concerns	Very low
recurrent myocardial infarction	Indirect evidence							
DI:STSI	0	Some concerns	Some concerns	Some concerns	Major concerns	No concerns	Major concerns	Very low
DI:DCI	0	Some concerns	Some concerns	Some concerns	Major concerns	No concerns	Major concerns	Very low
STSI:DCI	0	Some concerns	No concerns	Some concerns	Major concerns	No concerns	Major concerns	Very low
LVEF	Mixed evidence							
PI:CT	4	Some concerns	No concerns	No concerns	No concerns	Major concerns	Major concerns	Very low
DI:CT	12	Some concerns	Some concerns	Some concerns	No concerns	Major concerns	Major concerns	Very low
STSI:CT	6	Some concerns	Some concerns	Some concerns	No concerns	Major concerns	Major concerns	Very low
DCI:CT	5	Some concerns	No concerns	No concerns	No concerns	Major concerns	Major concerns	Very low
LVEF	Indirect evidence							
PI:DI	0	Some concerns	Some concerns	Some concerns	No concerns	Major concerns	Major concerns	Very low

PI:STSI	0	Some concerns	Some concerns	Some concerns	Major concerns	No concerns	Major concerns	Very low
PI:DCI	0	Some concerns	No concerns	No concerns	Major concerns	No concerns	Major concerns	Very low
DI:STSI	0	Some concerns	Some concerns	Some concerns	No concerns	Major concerns	Major concerns	Very low
DI:DCI	0	Some concerns	Some concerns	Some concerns	No concerns	Major concerns	Major concerns	Very low
STSI:DCI	0	Some concerns	Some concerns	Some concerns	Major concerns	No concerns	Major concerns	Very low
Adverse events	Mixed evidence							
PI:CT	3	Some concerns	No concerns	No concerns	No concerns	Major concerns	Major concerns	Very low
DI:CT	11	Some concerns	Some concerns	Some concerns	No concerns	No concerns	Major concerns	Very low
STSI:CT	6	Some concerns	Some concerns	Some concerns	No concerns	No concerns	Major concerns	Very low
DCI:CT	3	Some concerns	No concerns	No concerns	No concerns	No concerns	Major concerns	Very low
Adverse events	Indirect evidence							
PI:DI	0	Some concerns	Some concerns	Some concerns	Major concerns	Major concerns	Major concerns	Very low
PI:STSI	0	Some concerns	Some concerns	Some concerns	Major concerns	Major concerns	Major concerns	Very low
PI:DCI	0	Some concerns	No concerns	No concerns	Major concerns	Major concerns	Major concerns	Very low
DI:STSI	0	Some concerns	Some concerns	Some concerns	Major concerns	No concerns	Major concerns	Very low
DI:DCI	0	Some concerns	Some concerns	Some concerns	Major concerns	No concerns	Major concerns	Very low
STSI:DCI	0	Some concerns	Some	Some	Major concerns	No concerns	Major	Very low

			concerns	concerns			concerns	

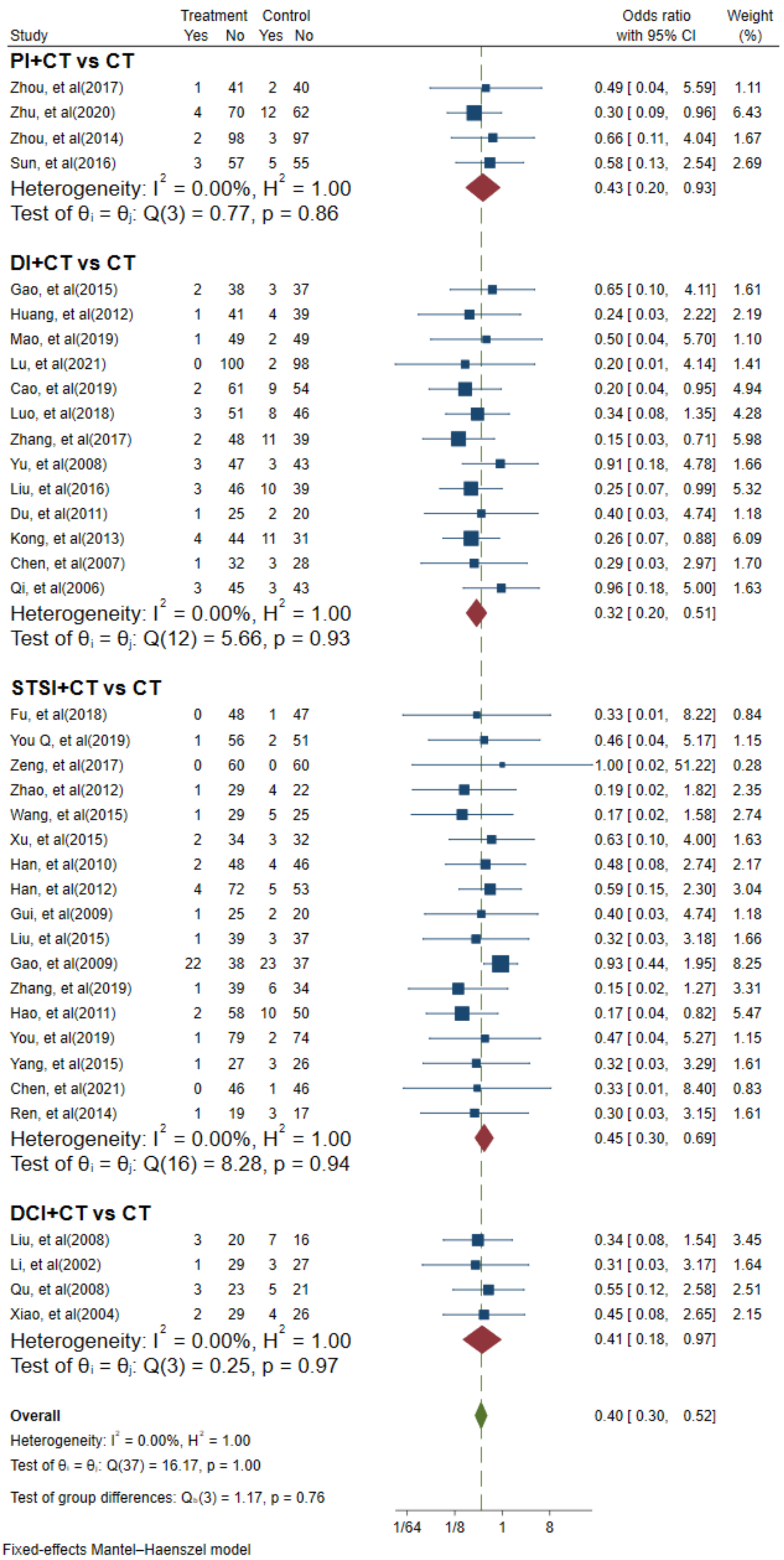

Supplementary figure F2 Forest plot of the effect of TCMi-ABC combined with conventional western medicine treatment on all-cause mortality in patients with AMI

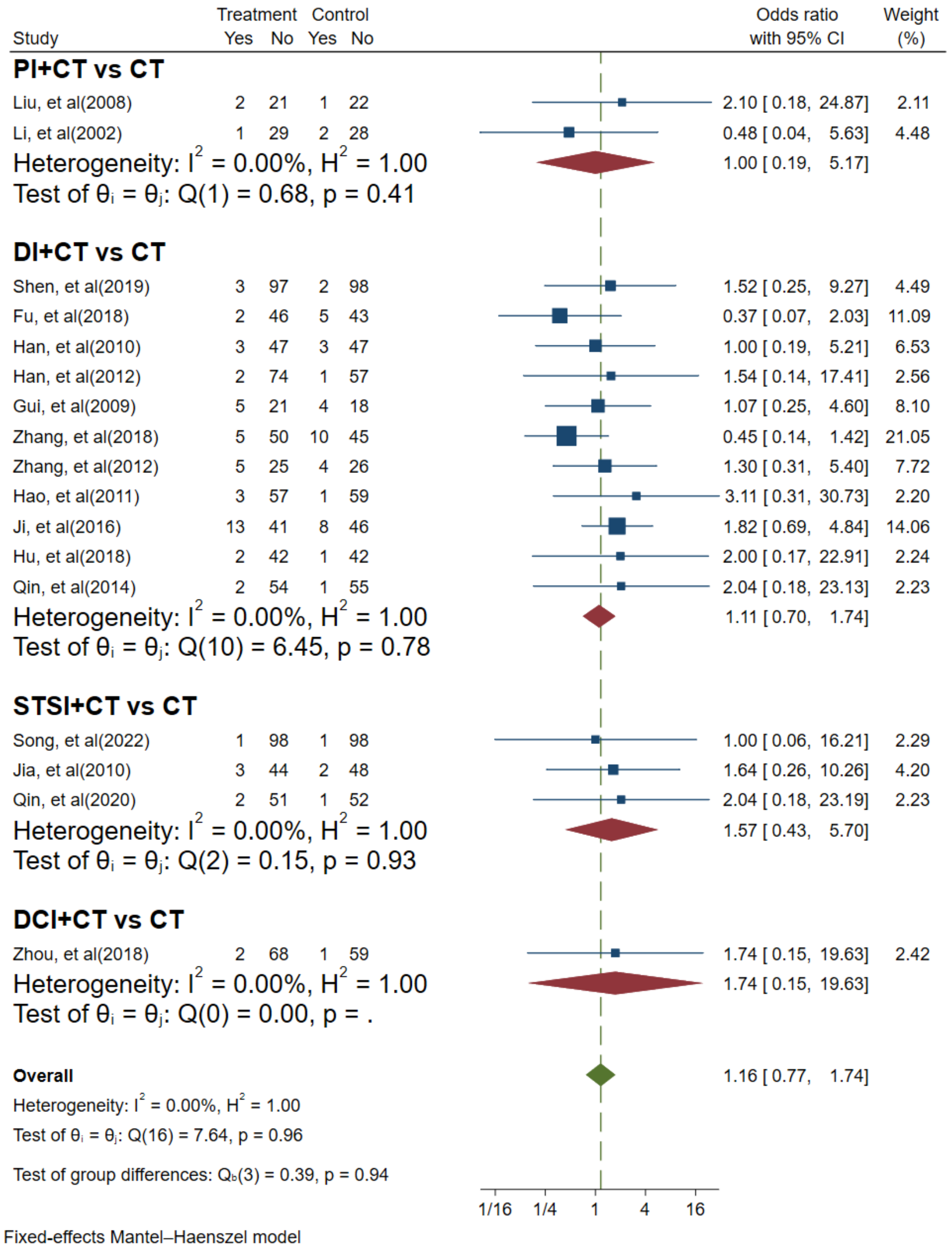

Supplementary figure F3 Forest plot of the effect of TCMi-ABC combined with conventional western medicine treatment on the incidence of bleeding events in patients with AMI

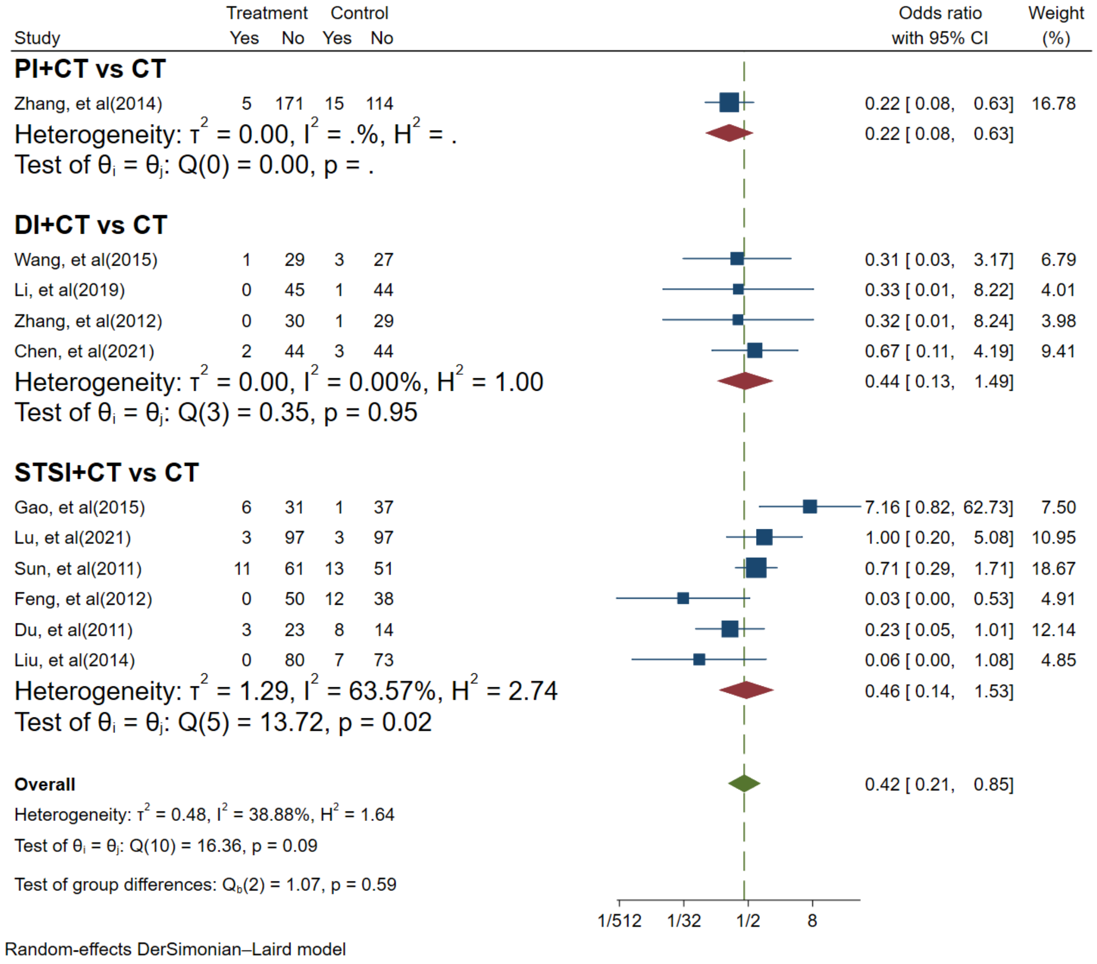

Supplementary figure F4 Forest plot of the effect of TCMi-ABC combined with conventional western medicine treatment on the incidence of malignant arrhythmia in patients with AMI

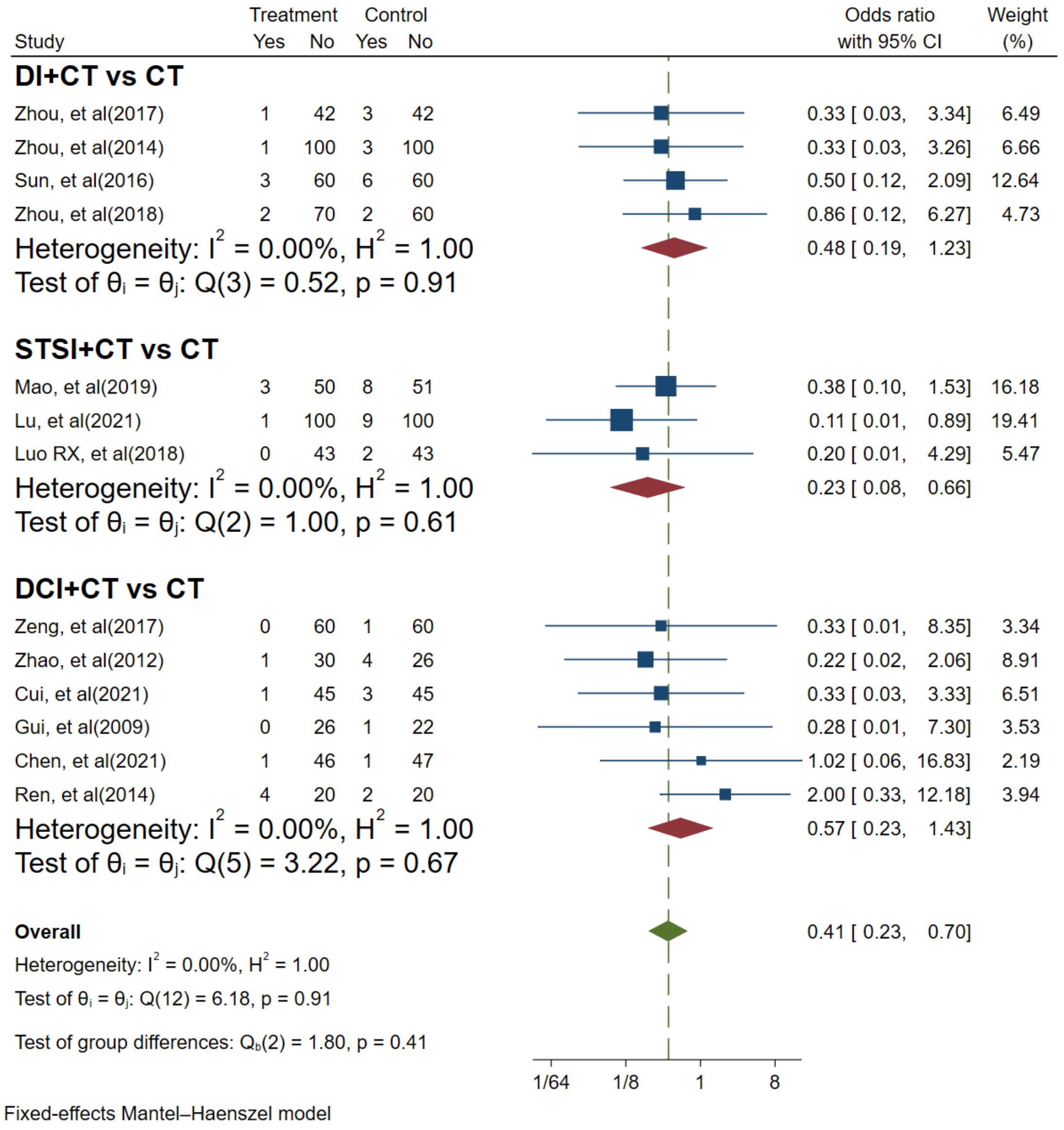

Supplementary figure F5 Forest plot of the effect of TCMi-ABC combined with conventional western medicine treatment on the incidence of recurrent myocardial infarction in patients with AMI

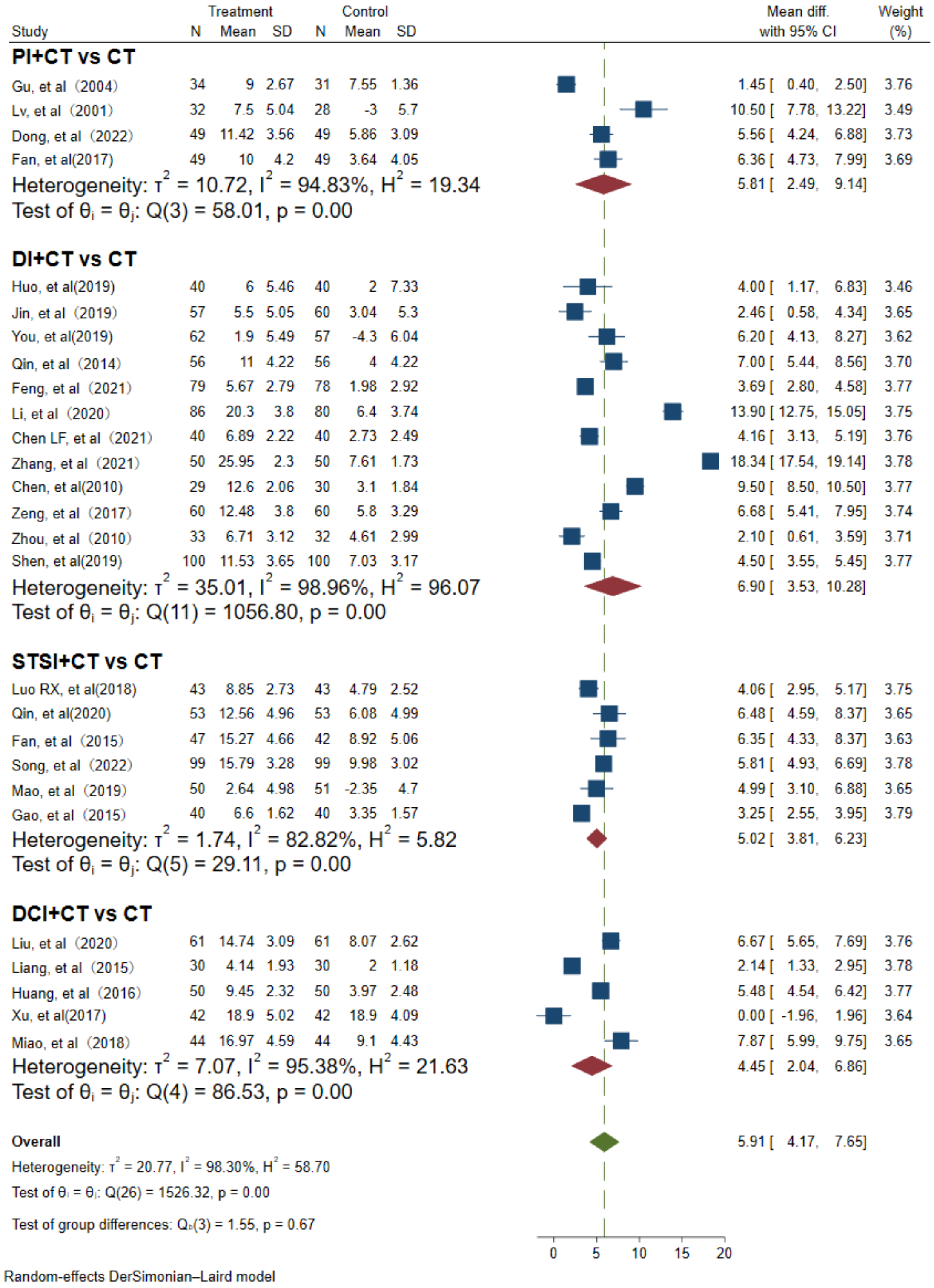

Supplementary figure F6 Forest plot of the effect of TCMi-ABC combined with conventional western medicine treatment on LVEF in patients with AMI

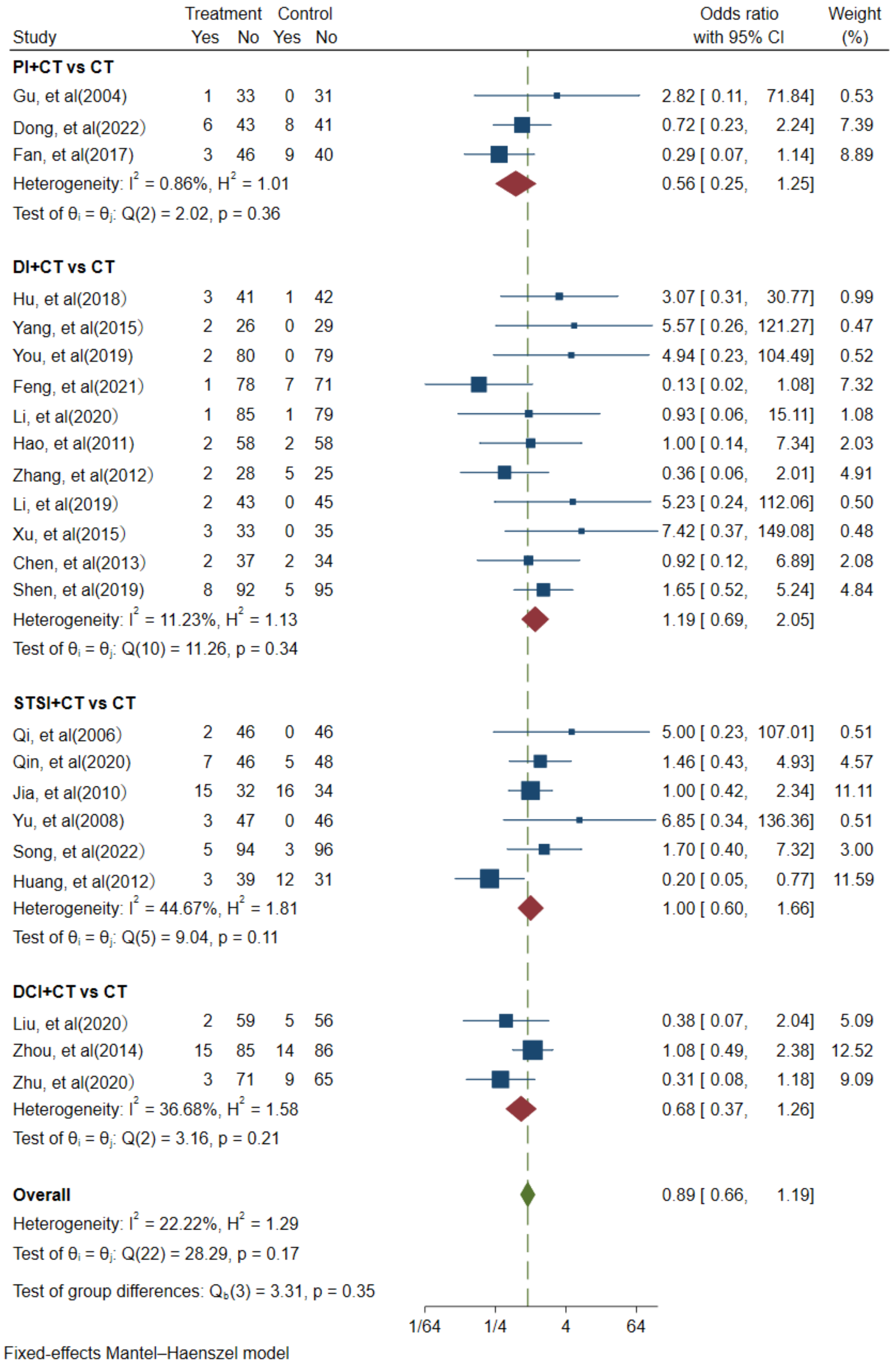

Supplementary figure F7 Forest plot of the effect of TCMi-ABC combined with conventional western medicine treatment on adverse events in patients with AMI

Supplementary table S14 Subgroup analysis of the incidence of malignant arrhythmia of AMI patients treated with STSI based on mean age, reperfusion therapy category, duration of treatment, and sample size

Grouping Criteria	Subgroups	n	I^2(%)	OR(95%*CI*)
mean age	＞59 years old	3	82.62	0.26(0.01，9.94)
	≤59 years old	3	6.54	0.58(0.28，1.21)
reperfusion therapy category	percutaneous coronary intervention	3	70.30	1.03(0.16，6.47)
	thrombolysis, or coronary artery bypass surgery	3	68.08	0.16(0.02，1.43)
duration of treatment	＞7 days	3	69.71	0.86(0.19，3.91)
	⩽7 days	3	65.18	0.16(0.01，1.69)
sample size	≥130	3	31.08	0.58(0.20，1.70)
	＜ 130	3	80.85	0.40(0.02，6.60)

Supplementary table S15 Subgroup analysis of LVEF based on mean age, baseline value of LVEF, sample size, duration of treatment, and reperfusion therapy category

Grouping Criteria	Subgroups	n	I^2(%)	MD(95%*CI*)
mean age	≤60 years old	14	96.23	5.83(4.21，7.44)
	＞60 years old	13	98.91	5.98(2.74，9.23)
baseline value of LVEF	＜40%	4	93.22	3.15(1.14，5.16)
	[40%, 45%)	13	98.79	6.35(3.33，9.37)
	⩾45%	10	97.37	6.49(3.92，9.06)
Sample size	＜ 100	14	94.65	4.74(3.29，6.19)
	≥100	13	98.80	7.11(4.20，10.02)
Duration of treatment	7 days	7	84.86	4.59(3.12，6.06)
	14 days	16	98.86	6.53(3.94，9.12)
	＞14 days	4	94.82	5.54(2.70，8.37)
reperfusion therapy category	thrombolysis	7	99.30	6.96(1.36，12.57)
	percutaneous coronary intervention	12	97.29	5.93(3.89，7.96)
	No reperfusion therapy	8	90.43	4.94(3.47，6.41)

Supplementary table S16 Pooled estimates of the sensitive analysis (case number ⩾ 80)(All-cause mortality, Incidence of bleeding events)

ACM (Left lower part)	Incidence of bleeding events (Right upper part)		
**DI+CT**	0.71 (0.13, 3.37)	-	1.09 (0.58, 2.10)
1.46 (0.61, 3.07)	**STSI+CT**	-	1.55 (0.33, 7.71)
1.02 (0.35, 2.99)	0.73 (0.25, 2.08)	**DCI+CT**	-
**0.45 (0.23, 0.77)**	**0.30 (0.18, 0.53)**	0.44 (0.17, 1.16)	**CT**

Note: Bold numbers in the table indicate a statistically significant difference between this group and the CT group ($P < 0.05$).

Supplementary table S17 Pooled estimates of the sensitive analysis (case number ⩾ 80)(Incidence of malignant arrhythmias, Incidence of recurrent myocardial infarction)

RMI (Left lower part)	MA (Right upper part)		
**DI+CT**	1.77 (0.01, 448.61)	-	0.20 (0.00, 13.68)
1.93 (0.15, 22.62)	**STSI+CT**	-	0.11 (0.00, 1.56)
0.76 (0.05, 6.32)	0.43 (0.05, 2.11)	**DCI+CT**	-
0.34 (0.03, 2.09)	**0.19 (0.03, 0.64)**	0.42 (0.13, 1.40)	**CT**

Note: Bold numbers in the table indicate a statistically significant difference between this group and the CT group ($P < 0.05$).

Supplementary table S18 Pooled estimates of the sensitive analysis (case number⩾

80)(LVEF, adverse events)

LVEF(Left lower part)	Adverse events (Right upper part)			
**PI+CT**	0.31 (0.03, 2.39)	2.98 (0.42, 28.79)	0.83 (0.09, 8.99)	0.44 (0.07, 2.61)
-1.49 (-8.12, 5.21)	**DI+CT**	0.92 (0.19, 4.98)	2.69 (0.46, 21.29)	1.45 (0.48, 5.25)
0.82 (-6.12, 7.93)	2.35 (-2.40, 7.01)	**STSI+CT**	2.47 (0.45, 19.75)	1.31 (0.48, 4.88)
0.91 (-6.52, 8.50)	2.48 (-2.91, 7.68)	0.15 (-5.49, 5.86)	**DCI+CT**	0.54 (0.11, 2.19)
5.94 (-0.06, 12.08)	**7.48 (4.54, 10.36)**	**5.14 (1.54, 8.71)**	**5.01 (0.65, 9.33)**	**CT**

Note: Bold numbers in the table indicate a statistically significant difference between this group and the CT group ($P < 0.05$).
